# Microglia at the blood brain barrier in health and disease

**DOI:** 10.3389/fncel.2024.1360195

**Published:** 2024-03-13

**Authors:** Meredith G. Mayer, Tracy Fischer

**Affiliations:** ^1^Division of Comparative Pathology, Tulane National Primate Research Center, Covington, LA, United States; ^2^Department of Microbiology and Immunology, Tulane University School of Medicine, New Orleans, LA, United States

**Keywords:** microglia, endothelial cells, neuroinflammation, BBB, stroke, systemic inflammation, blood-brain barrier

## Abstract

The blood brain barrier (BBB) plays a crucial role in maintaining brain homeostasis by selectively preventing the entry of substances from the peripheral blood into the central nervous system (CNS). Comprised of endothelial cells, pericytes, and astrocytes, this highly regulated barrier encompasses the majority of the brain’s vasculature. In addition to its protective function, the BBB also engages in significant crosstalk with perivascular macrophages (MΦ) and microglia, the resident MΦ of the brain. These interactions play a pivotal role in modulating the activation state of cells comprising the BBB, as well as MΦs and microglia, themselves. Alterations in systemic metabolic and inflammatory states can promote endothelial cell dysfunction, reducing the integrity of the BBB and potentially allowing peripheral blood factors to leak into the CNS compartment. This may mediate activation of perivascular MΦs, microglia, and astrocytes, and initiate further immune responses within the brain parenchyma, suggesting neuroinflammation can be triggered by signaling from the periphery, without primary injury or disease originating within the CNS. The intricate interplay between the periphery and the CNS through the BBB highlights the importance of understanding the role of microglia in mediating responses to systemic challenges. Despite recent advancements, our understanding of the interactions between microglia and the BBB is still in its early stages, leaving a significant gap in knowledge. However, emerging research is shedding light on the involvement of microglia at the BBB in various conditions, including systemic infections, diabetes, and ischemic stroke. This review aims to provide a comprehensive overview of the current research investigating the intricate relationship between microglia and the BBB in health and disease. By exploring these connections, we hope to advance our understanding of the role of brain immune responses to systemic challenges and their impact on CNS health and pathology. Uncovering these interactions may hold promise for the development of novel therapeutic strategies for neurological conditions that involve immune and vascular mechanisms.

## Blood–brain barrier function

**Figure 1:**
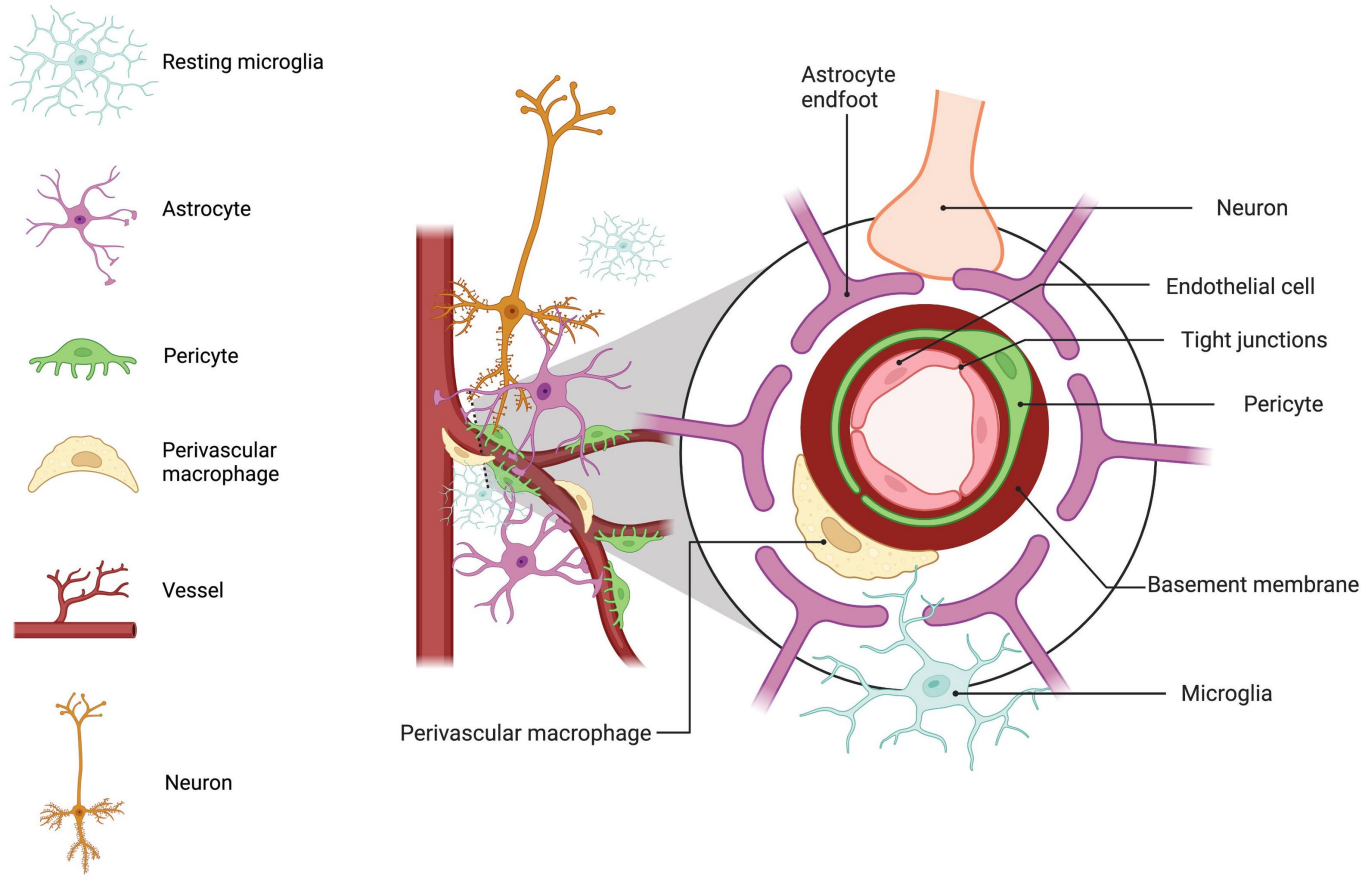
Blood brain barrier (BBB) diagram. BBB structure is displayed with a cross-section indicating the arrangement of the endothelial cell, pericyte, basement membrane, perivascular macrophage (MΦ), astrocyte end-foot, neuron, and microglia. Basal lamina or basement membrane shown in red provides support and structure for BBB. Pericytes are embedded in the basement membrane of the vasculature mediating vessel development (Vanlandewijck et al., 2018), blood flow (McCaffrey et al., 2007), astrocyte end feet polarization (de Oliveira et al., 2020), and prevention of BBB leakage (Chehade et al., 2002). Between the vascular basement membrane and glial limitans perivascular MΦs surveil for foreign antigens and regulate macromolecule movement (Sheikh et al., 2022). Astrocyte end feet surround the vasculature and basal lamina producing factors contributing to the maintenance of the BBB and TJ expression (Nielsen et al., 1997; Sobue et al., 1999; Manley et al., 2000; Alvarez et al., 2011; Song et al., 2018). The vasculature receives signaling from neurons in the surrounding area altering local blood flow (Kuchler-Bopp et al., 1999). The legend displays: microglia, light blue; astrocytes, lilac; pericytes, green; perivascular macrophage, yellow; blood vessel, red; neuron, orange. This figure created with BioRender.com.

The central nervous system (CNS) is a voracious consumer of energy and requires a constant and substantial supply of oxygen and glucose, as well as a means for removing detrimental byproducts associated with energy consumption. To meet these needs, the CNS comprises an extensive vascular network that delivers an uninterrupted flow of resources crucial to sustaining optimal function of the brain and spinal column. This vascular architecture also protects the brain by facilitating the removal of potentially harmful substances from the CNS compartment and preventing the ingress of neurotoxic factors from the peripheral blood. Known as the blood–brain barrier (BBB), this extraordinary and intricate biological construct stands as a highly selective and protective threshold that demarcates the juncture between the periphery and CNS (Figure 1).

The BBB is composed of specialized brain endothelial cells and astrocyte end feet that form contacts with the endothelial cells. The astrocyte end foot processes form rosette-like structures (Kacem et al., 1998) on the surface of the endothelial cells maintaining the resting potential and water permissibility through passive water channel, aquaporin 4 (Nielsen et al., 1997; Manley et al., 2000), and inward-rectifier Kir4.1 potassium channels (Song et al., 2018). Astrocytes generate an inductive tightening influence on the endothelium through the production of sonic hedgehog (Shh) (Alvarez et al., 2011) and basic fibroblast growth factor (bFGF) (Sobue et al., 1999) that assist in forming a restrictive BBB by promoting endothelial expression of tight junction (TJ) proteins, occludin, claudins, and zonula occludens (ZO). Astrocytes also produce α-dystrobrevin, which contributes to BBB integrity through cell adhesion and cytoskeletal organization (Lien et al., 2012). Together, these factors contribute to the formation and maintenance of the BBB by increasing expression of TJ proteins, that form a tight seal between endothelial cells and prevent the uncontrolled passage of substances between the peripheral blood and CNS. Additionally, several astrocytic-derived proteins, including bFGF, transforming growth factor-β (TGF-β), and neurotrophic factors support a restrictive BBB through up-regulation of various transporters and TJs (Boado et al., 1994; Igarashi et al., 1999; Kuchler-Bopp et al., 1999; Sobue et al., 1999; Lin et al., 2018; Wen et al., 2023) that limit passive transport of polar substances and enhances active transport of necessary nutrients into the CNS compartment and active efflux of toxic metabolites and other harmful substances.

Compared to other organs, brain endothelial cells have a higher expression of occludin, claudin-5, and ZO-1 (McCaffrey et al., 2007; Vanlandewijck et al., 2018) and an intact BBB is vital for maintaining brain homeostasis. Many neurodegenerative diseases are associated with reduced BBB integrity, including Alzheimer’s and Parkinson’s disease and amyotrophic lateral sclerosis (ALS), however, the mechanisms underlying impaired BBB function and integrity are not fully understood. Oxidative stress and metabolic dysfunction within cells comprising the BBB in the context of excessive and/or prolonged inflammation are posited to contribute significantly to BBB injury. Furthermore, metabolic stressors, such as hypercholesterolemia, have been reported to promote BBB disruption, with increased permeability and decreased claudin-5 and occludin transcription (de Oliveira et al., 2020). Similarly, untreated type 2 diabetes mellitus is associated with decreased expression of occludin (Chehade et al., 2002) and increased vascular permeability (Sheikh et al., 2022).

Unresolved inflammation can result in sustained and the production of multiple factors that contribute to decreased TJ expression and increased BBB permeability, including interleukin (IL)-1β, tumor necrosis factor alpha (TNF-α), IL-6, nuclear factor kappa B (NF-κB), matrix metalloproteinase (MMP)-9, advanced glycation end-products (AGEs), and reactive oxygen species (ROS) (Hofmann et al., 1999; Laflamme et al., 1999; Spranger et al., 2003; Hawkins et al., 2007; Niiya et al., 2012; Sajja et al., 2014; Aggarwal et al., 2015; Dobi et al., 2021; Gao and Bayraktutan, 2023). Oxidative stress, which arises when production of free radicals exceeds their neutralization, is a common complication of chronic inflammation and can have detrimental effects on the integrity of the BBB and further perpetuate inflammatory responses within the brain (Schreibelt et al., 2007; Zeng et al., 2017; Kayano et al., 2018; Uddin et al., 2021). Key contributing factors in the development of oxidative stress include the generation of AGEs and ROS, as a result of excessive aerobic glucose metabolism. These molecules activate the NF-κB pathway, which in turn promotes expression of MMP-9 (Schreck et al., 1991; Wang et al., 2011; Zhao et al., 2013; Luo et al., 2021). This enzyme breaks down the extracellular matrix, promoting BBB instability (Kim et al., 2021; Hu et al., 2022; Liu et al., 2022). NF-κB activation also results in upregulation of several cytokines, including IL-1β, TNF-α, and IL-6, all of which are closely associated with inflammation.

In the context of metabolic dysfunction and inflammation, endothelial cells forming the BBB exhibit heightened levels of adhesion and transmigration molecules, intercellular adhesion molecule (ICAM), vascular cell adhesion molecule (VCAM), E-selectin, and P-selectin (Close et al., 2013). Additionally, there is an increase in circulating chemoattractant molecules, like monocyte chemoattractant protein (MCP)-1 and IL-8 (Close et al., 2013). Notably, MCP-1, also known as chemokine ligand 2 (CCL2), plays a pivotal role in promoting BBB instability by facilitating the recruitment of monocytes/MΦs into CNS compartment and contributing to the reorganization of TJ and the actin cytoskeleton (Stamatovic et al., 2003). Taken together, the activation of oxidative and inflammatory pathways in cerebral endothelial cells provides a crucial route for translating peripheral inflammatory and oxidative stress signals through the BBB into the brain parenchyma. This mechanism underscores the intricate relationship between oxidative stress, inflammation, and BBB integrity, all of which are critical factors in the development and progression of neurodegenerative diseases like Alzheimer’s disease (AD).

## Microglia function

Microglia, the resident MΦ and principal immune cell of the CNS, play a multifaceted role beyond their conventional immune function. They contribute significantly to various aspects of brain function, including brain development, learning and memory processes, and the maintenance of CNS homeostasis. Microglia continuously survey the brain by extending long processes that allow them to assess changes in the microenvironment. The area of microglial surveillance is further increased through induction of thin filopodia that extend from the larger processes and dynamically extend and retract. This movement is facilitated by localized cyclic adenosine monophosphate (cAMP) and enables microglia to monitor changes in their microenvironment and quickly respond to molecular cues (Nimmerjahn et al., 2005; Bernier et al., 2019). In their role as primary immune cell of the CNS, they are crucial for maintaining a “clean” microenvironment by preventing the accumulation of cellular debris and metabolic waste products.

Importantly, microglia are not limited to immune functions alone. They also strengthen brain function through synapse organization and are key participants in the development and maintenance of neural circuitry by pruning of excess neuronal synapses (Paolicelli et al., 2011). Microglia also support neovascularization within the CNS by providing a structural framework for growing and developing vessels (Grossmann et al., 2002; Ginhoux et al., 2010; Dudiki et al., 2020; Mondo et al., 2020; Hikage et al., 2021). While not traditionally associated with the BBB, microglia do interact and communicate with brain vascular endothelial cells. This interaction becomes particularly relevant when considering the regulation of solutes, chemicals, and foreign antigens entering the brain parenchyma. While a healthy, intact BBB tightly restricts the movement of these substances into the CNS compartment, these, and other myeloid-responsive factors, such as cytokines, are not fully restricted from entering the CNS, which may compromise BBB integrity through stimulation of perivascular MΦs and microglia. Additionally, even metabolic factors, like glucose levels can impact microglial activity. Conditions, such as diabetes or hyperglycemia, can lead to increased microglial reactivity, marked by upregulation of various molecules and pathways associated with glucose transport and sensing and inflammation and oxidative stress (Hsieh et al., 2019; Iannucci et al., 2022; Vargas-Soria et al., 2023).

As innate immune cells, microglia function as mediators in response to brain stress and injury. This is evidenced by changes in microglial morphology and transcriptional profiles, which are influenced by molecular signals encountered during their surveillance (Davalos et al., 2005; Nimmerjahn et al., 2005; Bernier et al., 2019). Resting microglia typically display long, ramified processes; however, exposure to pro-inflammatory cytokines, such as IL-1β (Monif et al., 2016; Davis et al., 2018), TNF-α (Kuno et al., 2005; Lively and Schlichter, 2018), IL-6 (Krady et al., 2008; Garner et al., 2018), stimulate the retraction of these processes, allowing the cells to become “amoeboid” in their movement (Lehrmann et al., 1997), which is driven by several signaling molecules, including extracellular adenosine triphosphate (ATP), dead cells, and cellular debris (Davalos et al., 2005; Nimmerjahn et al., 2005). Notably, inflammatory conditions may lead to a positive feedback loop in microglia, resulting in the secretion of proinflammatory mediators (Kuno et al., 2005), which can be neurotoxic and worsen existing damage (Zhang et al., 2004; Li et al., 2021). Polarization of microglia to a reactive state can have pathological implications. The underlying cause of immune polarization is not clear but may involve the continued presence of “on” signaling mediators, such as proinflammatory factors, and/or the absence of “off” signaling activity through CD200, CD47 (Hoek et al., 2000; Wright et al., 2001), and/or CX3C motif chemokine receptor 1 (CX3CR1) (Cardona et al., 2006). Identifying the processes involved in chronic microglial activation is crucial in understanding neurodegenerative and neuropathological diseases, as prolonged inflammation often plays a role in these conditions (McGeer et al., 1987, 1988; Cagnin et al., 2001a,b; Puntener et al., 2012; Chiot et al., 2020; Zelic et al., 2021; Rutkai et al., 2022).

## Microglia and brain endothelial cells

Microglia originate from yolk-sac and begin to populate the CNS early in embryonic development and before the formation of the BBB (Santos et al., 2008; Hristova et al., 2010; Rigato et al., 2011; Swinnen et al., 2013). During this early stage, microglial seeding of the CNS plays a crucial role in influencing the direction of vascular growth in the developing brain (Ginhoux et al., 2010). Microglia are also known to closely follow vascular sprouts and establish associations with endothelial cells (Rezaie et al., 1997; Grossmann et al., 2002; Monier et al., 2006). One intriguing theory suggests that colonizing microglia are guided by a fractalkine gradient, which is sensed through the microglial fractalkine receptor, CX3CR1 (Mondo et al., 2020), which helps guide microglia along the developing vasculature. Interestingly, during this developmental phase, astrocytes have not yet encapsulated the vasculature, allowing microglial processes to contact the endothelium, facilitating more direct interactions between microglia and the blood vessels (Mondo et al., 2020). Around 18–24 gestational weeks, approximately 38% of microglia are seen in close proximity to blood vessels, with their soma located about 30 μm from the vessel (Mondo et al., 2020). This proximity allows for more direct interactions between microglia and the endothelial cells compared to the mature brain, where such interactions are more transient under resting surveilling conditions.

It is worth noting that there are distinctions between capillary-associated and parenchymal microglia, however, parenchymal microglia can transition to capillary-associated microglia (Bisht et al., 2021). Capillary-associated microglia (CAMs) are characterized by expression of CX3CR1 and their interactions with purines released from pannexin-1 (PANX1) channels. The coupling of PANX1 with P2Y purinoceptor 12 (P2RY12) in resting microglia helps maintain optimal capillary diameter, cerebral blood flow, and vascular responsiveness (Bisht et al., 2021). Studies employing advanced imaging techniques, such as confocal laser scanning microscopy and immune-electron microscopy, of CX3CR1^tdTomato^ microglia reporter mice have demonstrated that microglial processes cover approximately 15% of the endothelial cell surface. These processes express the P2RY12 receptor and make direct contact with smooth muscle cells, pericytes, and endothelial cells of the vasculature (Császár et al., 2022).

CAMs also participate in the regulation of cerebral blood flow through CX3CR1 and P2RY12 activity. A retinal study found evidence that microglia fractalkine-CX3CR1 signaling is vasoconstrictive (Mills et al., 2021). Additionally, reactive oxygen species (ROS), secreted in large amounts by activated microglia, can activate the Rho-kinase pathway, leading to pericyte contraction and inhibition of vasodilation, thus promoting vasoconstriction (Hartmann et al., 2021). Microglial P2RY12 can also be stimulated by purinergic signaling of cells comprising the neurovascular unit, inducing changes in cerebral blood flow through vasodilation (Császár et al., 2022). This aspect of microglia-mediated blood flow signaling was briefly reviewed by Dufort et al. (Dufort et al., 2022).

Endothelial cells of the BBB also respond to injury and inflammation, which would, presumably, influence microglial activation. Endothelial cells possess immune capabilities, including recruitment of immune cells into the CNS compartment via toll-like receptors (TLR) (Nagyőszi et al., 2010) and chemokine receptors (Johnson and Jackson, 2010; Salsman et al., 2011; Wilhelmsen et al., 2012). For example, when activation of endothelial TLR2 induces production of inflammatory mediators and chemoattractants, such as IL-6, granulocyte colony stimulating factor (G-CSF), and IL-8 (Wilhelmsen et al., 2012), which can further immune responses by acting on neighboring microglia and perivascular macrophages. Importantly, damage to the endothelial cell layer and/or inflammatory stimuli from the peripheral blood in circulation can also prompt microglia activation and migration to the vasculature (Haruwaka et al., 2019).

At the brain vasculature, microglia exhibit a critical balance between protective and detrimental roles in neuroinflammation and BBB integrity. Recent evidence of microglial expression of the TJ protein, claudin-5, at the vasculature was observed in a systemic lupus erythematosus (SLE) mouse model (Haruwaka et al., 2019). Following induction with lipopolysaccharide (LPS) or interferon alpha (INF-α), c-c chemokine receptor 5 (CCR5) facilitated the movement of microglia to the vasculature (Haruwaka et al., 2019). The directed movement of microglia toward injured or stimulated vasculature underscores a protective mechanism aimed at shielding the brain from neurotoxic factors; however, activated microglia can increase BBB permeability and vascular leakage (Haruwaka et al., 2019), potentially through the release of proinflammatory mediators and generation of ROS. Such activities highlight the dual nature of microglial engagement with the BBB, where on one side, they aid in the recovery of BBB permeability by limiting the infiltration of peripheral factors into the CNS parenchyma, yet on the other, they contribute to BBB dysfunction.

The pathological impact of activated microglia on the BBB is suggested through the impact of inflammatory factors on endothelial TJ protein expression, which are increased in the context of microglial activation. Proinflammatory cytokines, TNF-α and IL-1β, as well as ROS, have been implicated in the disruption of BBB integrity, marked by downregulation of TJ proteins, occludin, claudin-5, and ZO-1 (Nishioku et al., 2010; Kacimi et al., 2011; Shigemoto-Mogami et al., 2018). Experimental models have demonstrated that primary murine microglia, when stimulated with amyloid beta before co-culture with mouse brain endothelial cells, induce BBB damage (Mehrabadi et al., 2017). This damage was mediated through the release of TNF-α and nitric oxide (NO), which diminished the expression of TJ protein (Mehrabadi et al., 2017). Conversely, unstimulated microglia appear to bolster the expression of TJ proteins within co-cultured endothelial cells, demonstrating a protective or restorative role of microglia in BBB integrity (Mehrabadi et al., 2017). Adding complextity to BBB-microglia interaction, a study by Krasnow et al., revealed that murine brain microvascular endothelial cells exposed to IL-1β before co-cultured with microglia, amplifies inflammatory gene expression in microglia, as compared to IL-1β exposure of isolated microglia (Krasnow et al., 2017). This suggests a bidirectional communication mechanism between endothelial cells and microglia (Krasnow et al., 2017), further emphasizing the intricate relationship between neuroinflammation, microglial activation, and BBB functionality.

This body of evidence collectively underscores the nuanced and multifaceted role of microglia in neuroinflammation and BBB regulation. Understanding the balance between protective and harmful microglial functions is essential for developing therapeutic strategies targeting neurodegenerative diseases and systemic inflammatory conditions. The interplay between microglia, TJ proteins, and inflammatory mediators offers potential avenues for intervention aimed at preserving or restoring BBB integrity. Accordingly, the interaction between cells of the BBB and microglia is an emerging field of interest, particularly in the context of systemic diseases and viral infections that indirectly impact immune responses in the brain through endothelial and microglia cell activation. This review delves into the complex interactions at the BBB among microglia, endothelial cells, and other vascular components, alongside their engagement with peripheral blood. We also highlight critical knowledge gaps that need to be bridged to enhance therapeutic strategies focused on rejuvenating brain health.

## Stroke

The brain is highly vascularized and particularly vulnerable to stroke, which occurs when the blood supply in the brain is disrupted. Stroke is a complex neurovascular disease that is often associated with comorbidities, such as elevated glucose and/or low-density lipoprotein cholesterol levels, hypertension, atherosclerosis, and natural aging (Tsao et al., 2023). This multifaceted predisposition for stroke can be attributed, at least in part, to the activation of endothelial cells, which undergo a transformation from an anticoagulant to procoagulant phenotype. This transformation involves increased or *de novo* expression of adhesion molecules, including E-selectin, P-selectin, intracellular adhesion molecule-1 (ICAM-1), and vascular cell adhesion molecule-1 (VCAM-1) (Knottnerus et al., 2009). It is increasingly clear that the initiation of strokes can be traced back to events occurring within the blood and at the endothelial cell layer of the BBB.

Ischemic or hemorrhagic stroke occurs when the continuous flow of blood in the brain is interrupted suddenly, due to a blocked or ruptured artery, respectively. Cell death can result as a consequence of the damage caused by the rupture and subsequent bleeding in the brain, as well as the disrupted blood flow that prevents the delivery of oxygen and vital nutrients to the brain. Neuroinflammation in the context of stroke is multifactorial and includes endothelium.

Endothelial cell injury within the stroke site and surrounding area triggers a series of molecular signaling events and protein expression that stimulates microglia, which attempt to mitigate the damage caused by the stroke. Inflammation-associated transcripts may be enriched in endothelial cells following a stroke, as observed in a middle cerebral artery occlusion (MCAO) and reperfusion mouse models (Arbaizar-Rovirosa et al., 2023). This enrichment corresponds with increased cytokine and chemokine activity and immunoglobulin Fc-gamma receptor I complex binding that may further inflammation (Arbaizar-Rovirosa et al., 2023). For example, signaling through the chemokine receptor, CCR2, or Fc receptor promote inflammation through upregulation of the IL-6 pathway, leading to activation of signal transducer and activator of transcription 3 (STAT3) by ischemic endothelium (Arbaizar-Rovirosa et al., 2023). The proinflammatory transcriptional shift and BBB breach prompts microglia to migrate to the area of injury. Within hours of a stroke or modeled laser-ablated vessel, microglia begin their journey toward the affected region (Ahn et al., 2018; Lubart et al., 2021; Boghozian et al., 2023), which is prompted by a variety of signaling molecules, including fractalkine (Cao et al., 2019) and the purine, ATP (Davalos et al., 2005), extracellular peroxiredoxin (prx6) (Kuang et al., 2014), several Rho guanosine triphosphate hydrolyases (GTPases), like Rac, Cdc42, and Rho (Choi et al., 2011), CXCL12 (Huang et al., 2017), and apoptosis signal-regulating kinase 1 (ASK1) (Cheon et al., 2017). Importantly, irrespective of endothelial activation, CAMs respond to severely reduced blood flow, shifting to an activated state with morphological changes that support their migration (Masuda et al., 2011). Accumulating microglia in the periinfarct region exhibit diverse activation states with alternatively activated, phagocytic microglia, as well as classically activated microglia, characterized by upregulation of proinflammatory mediators (Hu et al., 2012; Huang et al., 2017). The microglial responses are designed to engulf cell debris, mediate repair, and reduce the neurotoxic effects of localized necrosis and infiltrating blood components, but may not fully resolve injury, which may promote a perpetuating cycle of microglial activation and BBB injury. Indeed, a non-resolved stroke is associated with persistent secondary inflammation, with profound impacts on pre-existing neurological conditions or subsequent brain injury.

Ischemic stroke, the most common type of stroke, can result from various factors, including blood clots (thrombi) and vascular plaques. Ischemic events result in two distinct regions of injury: the infarct core, which represents irreversibly damaged tissue (Peerlings et al., 2023), and the penumbra, where tissue damage may be reversible. Beyond the penumbra, the infarct region undergoes rapid necrosis, due to the lack of oxygen (Bandera et al., 2006), resulting in a hypoxic environment. Hypoxia stabilizes the oxygen-regulated alpha subunit of hypoxia inducible factor-1 (HIF-1α), which translocates to the nucleus, where it binds with the constituently expressed beta subunit of HIF-1 (HIF-1β), forming the transcription factor, HIF-1. HIF-1 binds hypoxia response element (HRE) (Rashid et al., 2019) in promoter regions of target genes, influencing angiogenesis, cell proliferation, erythropoiesis, and cell metabolism (Hong et al., 2020), as well as a initiating inflammatory responses and compromising BBB integrity. HIF-1α stabilization/upregulation is associated with increased expression of vascular endothelial growth factor (VEGF) (Mu et al., 2003; Shen et al., 2018; Zhang et al., 2018), glucose transporter 1 (GLUT1) (Lum et al., 2007; Zhong et al., 2010), and multiple chemokines (Arbaizar-Rovirosa et al., 2023) in brain endothelial cells (Hong et al., 2020) along with other transcriptional changes. The temporal sequence and regulation of HIF-1-dependent downstream proteins play a crucial role in the pathogenesis and recovery of stroke, potentially affecting the balance between inflammatory and angiogenetic responses.

Innate immune responses within the CNS are also impacted by HIF-1α stabilization downstream of transcriptional activities of HIF-1. Oxygen and glucose deprivation lead to the stabilization and/or upregulation of microglial HIF-1α (Xu et al., 2023), which, in turn, results in increased autophagy, TLR4, IL-1β, and IL-18 expression, and NLR family pyrin domain containing 3 (NLRP3) inflammasome formation, suggesting HIF-1 contributes to the proinflammatory phenotype of microglia after a stroke event (Guo et al., 2009; Jiang et al., 2020). While this is neuroprotective during the early stages of a stroke, sustained and/or expanded microglial activation can have detrimental consequence with prolonged neurotoxicity.

Microglia contribute significantly to proinflammation early after the onset of stroke (Li et al., 2021). Initial signaling events that further BBB breakdown or dysfunction after stroke are not completely clear, but likely result from multiple simultaneous events during the initial barrier disruption. Endothelial cells may mediate early inflammatory responses through expression of proinflammatory factors, like IL-1β (Iannucci et al., 2022), that activate microglia. Studies in mice lacking endothelial P-selectin glycoprotein ligand-1 (PSGL-1) and ICAM-1 demonstrate a reduction in activated microglia in the brain parenchyma, indicating a bidirectional communication of inflammation activation stemming from the endothelium (Atangana et al., 2017).

In addition to the acute injury, inflammation after stroke contributes to secondary cell injury, mediated, at least in part to NLRP3 inflammasome formation in microglia, MΦs located within the perivascular space, and endothelial cells themselves (Chen et al., 2019; Bellut et al., 2021). Inflammasome activation initiates a cascade of inflammation that perpetuates proinflammatory responses by myeloid and endothelial cells, which can be cytotoxic. For example, following ischemic stroke, microglial expression of TNF-α induces endothelial necroptosis in a rat model (Chen et al., 2019). Importantly, endothelial inflammasome activation is a key factor in BBB disruption and endothelial cell death after stroke, which may be triggered by microglial secretion of IL-1β (Bellut et al., 2021). This may point to the receptor for IL-1β, IL-1R1, as a viable therapeutic target for reducing BBB damage after ischemic reperfusion injury (Pan et al., 2023).

The inflammasome inflammatory cascade may be further intensified by HIF-1α feed-back loops with cytokines like IL-6, which is upregulated in proinflammatory conditions and with HIF-1α stabilization (Xing and Lu, 2016). This suggests that exacerbated inflammatory responses can persist even after the restoration of oxygen to the area of injury, as high levels of proinflammatory cytokines can re-stabilize HIF-1α, perpetuating the transcriptional signaling cascade (Abdi Sarabi et al., 2022).

Because microglia have varied effects on BBB recovery and repair after stroke, attempts to remedy excessive inflammation and secondary injury by inhibiting or depleting microglia populations, have yielded mixed results (Xing et al., 2018; Haruwaka et al., 2019; Zille et al., 2019). Pharmacologically induced depletion of resident microglia with tamoxifen and diphtheria toxin prior to the induction of ischemic stroke in a mouse model resulted in a decreased infarct volume and levels of pro-inflammatory factors (Li et al., 2021). Conversely, administration of the colony stimulating factor-1 receptor (CSF-1R) inhibitor, PLX3397, in a mouse model of stroke eliminated microglia, resulting in increased infarct size, neuronal signaling dysregulation, and cell death, which was reversed markedly by microglial repopulation (Szalay et al., 2016). A separate study employing the tyrosine kinase inhibitor, ki20227, with activity on CSF-1R, to inhibit microglial proliferation exacerbated microglial activation and neuronal injury after transient global cerebral ischemia (Hou et al., 2020). Temporarily depleting microglia with liposome-encapsulated clodronate injected intracerebrally lasted 3 days with reappearance of microglia after 5 days. The model displayed increased proinflammatory cytokine levels and damaged blood vessel integrity (Han et al., 2019). Together, these studies demonstrate microglial depletion is not a viable strategy for recovery after stroke and emphasizes the significance of microglia in recovery. Importantly, stimulation of CSF-1R with its cognate ligand, macrophage colony stimulating factor (M-CSF), is a critical factor in microglial function and promotes M2-like activation, which is key for resolving inflammation and tissue repair. Due to the complexity of microglial function in maintaining brain homeostasis and roles in injury response and repair, beneficial strategies that eliminate microglia or prevent interconversion of activation states seem unlikely.

Interestingly, inhibition of microglial activation and matrix metalloproteinases (MMPs) with minocycline reduced reperfusion injury (Liu et al., 2012; Yang et al., 2015). In rats assessed 2–4 weeks after ischemic injury, minocycline improved perfusion, reduced BBB permeability with higher levels of TJ proteins, and decreased the frequency of proinflammatory microglia, shifting their activation to an M2-like, or anti-inflammatory, phenotype with upregulation of transforming growth factor beta (TGF- β) and IL-10 and decreased TNF-α and IL-1β (Yang et al., 2015). Additionally, a transient MCAO CX3cr1-Cre mouse model with conditional knock-in overexpression of the chloride transmembrane transporter, Swell1, resulted in anti-inflammatory microglial activation and reduced brain injury (Chen et al., 2023). Brain and serum from Cre-Swell1 mice had lower levels of pro-inflammatory factors IL-1β, IL-6, macrophage inflammatory protein (MIP) 1β, TNF-α, and IFN-γ and increased levels of anti-inflammatory, IL-4 and IL-10, as compared to control animals (Chen et al., 2023). Further exploration demonstrated that Swell1 overexpression in a mouse microglial cell line, BV2 cells, cultured in a hypotonic environment to activate chlorine channels, activated cAMP response element-binding protein (CREB) and forkhead box O3 (FOXO3a) transcription factors and the negative regulator of the NLRP3 inflammasome, WNK lysine deficient protein kinase 1 (WNK1) (Chen et al., 2023). This study revealed that chlorine sensing signal pathways promte an anti-inflammatory responses by microglia, reducing injury and inflammation after stroke. Similarly, using a heterogeneous CX3cr-cre and loxP flanking site transgenic mouse model upregulating zinc finger E-box binding homeobox 1 (ZEB1) in microglia reduces CNS inflammation and neutrophil infiltration into the brain after transient MCAO (Li et al., 2018). ZEB1 regulates the development of the immune system and modulates cell differentiation (Funahashi et al., 1993; Arnold et al., 2012). Targeted microglial expression of ZEB1 in this model had reduced vascular injury, as suggested by less Evans blue extravasation into the brain, as compared to wildtype mice with transient MCAO (Li et al., 2018).

Typical of myeloid responses to injury, microglia demonstrate varied functions and activation states following stroke (Hu et al., 2012; Huang et al., 2017). A subpopulation of arginase 1 (Arg1)-expressing microglia exhibits an anti-inflammatory phenotype, with expression of IL-10 and TGF-β, and promotes recovery from stroke injury (Li et al., 2022). Notably, deletion of Arg1+ microglia promotes neuroinflammation in stroke models (Li et al., 2022), emphasizing the importance of reducing neuroinflammation and balancing microglia activation in recovery. Intranasal treatment of salvinorin A, a highly selective non-opioid kappa opioid receptor agonist, reduced neuroinflammation and BBB permeability in transient MCAO mice (Misilimu et al., 2022). Animals sacrificed 5 days after transient MCAO and treatment with salvinorin A demonstrated increased density of microglia near the infarction in the cortex and corpus striatum but with fewer microglia expressing CD16, a marker for pro-inflammatory microglia/macrophages in the cortex (Misilimu et al., 2022). These findings suggest that dampening pro-inflammatory microglial activation is beneficial for restoring BBB integrity and may improve recovery following stroke (Xing et al., 2018; Chen et al., 2022; Kuo et al., 2023; Liao et al., 2023). Administration of tissue plasminogen activator (tPA), which restores blood flow by dissolving blood clots, is considered the ‘gold standard’ for ischemic stroke and has saved the lives of countless lives and improved patient outcomes. Animal studies, however, have shown tPA can increase recruitment of peripheral immune cells to the site of injury that can contribute to secondary disruption of BBB and ischemic-related hemorrhagic bleeds of peripheral blood entering through the disrupted BBB (Fanne et al., 2010; Zhang et al., 2014). This may be averted through co-treatment with IFN-β, which has been shown to expand the Arg1+ microglial subset and reduce infarct volume and BBB disruption (Kuo et al., 2023).

## Diabetes mellitus

Diabetes mellitus (DM), commonly referred to as diabetes, is a chronic and serious health condition characterized by poor control of blood glucose levels. It arises from insufficient insulin production and/or impaired cell responses to insulin, resulting various adverse effects throughout the body, including the brain. Cognitive decline is a significant comorbidity of disease, particularly among individuals over the age of 65 years (Cheng et al., 2012). Notably, diabetes increases the risk for development of AD, vascular dementia (VD), and mild cognitive impairment (MCI) (Cheng et al., 2012). The mechanisms underlying diabetes-associated cognitive decline are not completely clear but are associated with neurodegeneration and macro- and microvascular injury (Qiu et al., 2014). Neuropathology investigations confirm an increased burden of cerebrovascular injury in the context of diabetes, as well as pathological changes associated with the perivascular space and surrounding parenchyma (Nelson et al., 2009; Abner et al., 2016).

Hyperglycemia, or elevated blood glucose levels, contributes to endothelial dysfunction, adversely affecting BBB integrity and triggering inflammatory responses in microglia. Additionally, increased cellular uptake of glucose leads to heightened mitochondrial respiration, resulting in elevated ROS production that can further inflammation and cell injury (Li et al., 2015; Arcambal et al., 2019). For example, studies conducted in murine b.End.3 endothelial cells have shown that hyperglycemia influences redox enzymes and oxidative stress, inducing the activation of proinflammatory signaling pathways NF-κB, c-Jun N-terminal kinase (JNK), extracellular signal-regulated kinase (ERK), and phosphoinositide 3-kinase (PI3K) (Arcambal et al., 2019). Hyperglycemia also contributes to oxidative stress through increased glucose metabolism. Elevated blood glucose in DM is a consequence of impaired production of or response to insulin, which facilitates glucose uptake. This results in abnormal metabolism of carbohydrates and increased glucose levels, which, in turn, can promote increased intracellular glucose metabolism, leading to oxidative stress (Ding et al., 2007), kallikrein-bradykinin activation (Campbell et al., 2010), and secretion of inflammatory cytokines (Nishikawa et al., 2000).

Excess glucose in circulation interacts with the microvascular and endothelial dysfunction caused by hyperglycemia is strongly associated with inflammatory signaling and oxidative stress. Endothelial cells exposed to high glucose levels induce expression of proinflammatory cytokines TNF-α and IL-6, as well as MMPs 2 and 9 (Vittal Rao et al., 2021). *In vitro* evidence has shown that high-glucose-induced calcium (Ca^2+^) secretion leads to fragmented mitochondria, resulting in increased ROS production and ERK 1/2 activation (Yu et al., 2011). While these data support the notion that high glucose contributes to reduced BBB integrity, at least in part through endothelial injury, there are conflicting reports on the endothelial barrier or TJ protein expression in the context of hyperglycemia. One study that utilized the Ins2^AKITA^ mouse model of type 1 diabetes showed no appreciable difference in BBB integrity, as compared to wildtype animals (Mäe et al., 2018). Conversely, leptin receptor deficient db/db mouse model of type 2 diabetes demonstrated increased BBB permeability, as compared to db/+ control mice (Yu et al., 2019). These seemingly conflicting reports may reflect differences in diabetes modeling. The Ins2^AKITA^ mouse has a single point mutation that causes misfolding of the insulin protein, resulting in the death of insulin-producing pancreatic β cells and reduced insulin secretion without significant weight gain. The db/db mouse contains a mutation in the leptin receptor, which impairs its normal function regulating appetite, leading to obesity and insulin resistance. While both models develop worsening disease with age, the db/db mouse may experience more severe vascular pathology with added weight-related comorbidity. It is important to note, however, that other *in vitro* and *in vivo* investigations suggest high glucose levels can directly impact BBB integrity through reduced TJ proteins. A study examining b.End.3 cells exposed to high glucose levels *in vitro* demonstrated a dose-dependent increase in permeability, with increased HIF-1α and decreased expression of the TJ proteins, ZO-1 and occludin (Yan et al., 2012). Additionally, a streptozotocin (STZ)-induced type 1 diabetes rat model, which also results in reduced insulin through β cell loss, was reported to have showed decreased endothelial ZO-1 and occludin and increased MMP activity in blood (Hawkins et al., 2007), suggesting changes in vascular permeability can be a direct consequence of high blood glucose.

Poor vascular health in diabetes patients is associated with an increased risk for stroke and experience worse stroke outcomes than non-diabetic patients (Akhtar et al., 2019; Lau et al., 2019). STZ-induced diabetic mice fed a high fat diet showed increased vascular leakage following MCAO, as compared to control mice (Abdul et al., 2021). This may be due to impaired endothelial function in diabetes (Luchsinger et al., 2001; Secrest et al., 2013), which may be further complicated by chronic activation of microglia around the vasculature and parenchyma. Microglia activation following ischemic events is exacerbated in hyperglycemic conditions with increased expression of proinflammatory mediators (Jackson et al., 2020; Abdul et al., 2021; Jackson-Cowan et al., 2021; Iannucci et al., 2022). Chronic pre-existing inflammation and imbalances in ROS/antioxidant production worsen stroke outcomes in diabetic patients and animal models (Tureyen et al., 2011; Akhtar et al., 2019; Lau et al., 2019; Bahader et al., 2021) with increased microglia activation and inflammation (Jackson et al., 2020; Jackson-Cowan et al., 2021). Some studies have explored antioxidant protection as a potential strategy for preventing endothelial cell injury induced by high glucose, exploiting the action of the transcription factor, nuclear factor erythroid 2-related factor (Nrf2). Nrf2 plays a pivotal role in stimulating the expression of antioxidant enzymes, metabolizing free radicals, and inhibiting inflammation (Itoh et al., 1997; Wu et al., 2012). Under homeostatic conditions, Nrf2 is bound to Kelch-like ECH-associated protein 1 (Keap1) and targeted for degradation through ubiquitination (McMahon et al., 2006). When oxidative stress conditions arise, the interaction between Nrf2 and Keap1 is disrupted, liberating Nrf2 to bind antioxidant response elements in the DNA. This leads to an increase in the activity of the glutathione-dependent enzymes, glutathione reductase and glutamate-cysteine ligase, and the glutamate/cystine antiporter, which are critical in the production of reduced glutathione, which plays a vital role in neutralizing ROS (Lee et al., 2003; Bell et al., 2011). Notably, Nrf2 provides an antioxidant response that is activated with both hyperglycemia endothelial cell *in vitro* models and an *in vivo* high fat diet mouse model (Ungvari et al., 2011). Hyperglycemia triggers an oxidative stress response that prompts Nrf2 to regulate antioxidant genes, detoxifying ROS. Interestingly, even under hypoglycemic conditions, Nrf2 expression is activated; however, prolonged hypoglycemia can reduce Nrf2 activity (Sajja et al., 2014, 2015).

Poorly managed therapeutic insulin can cause diabetic patients to experience broad fluctuations in glucose concentrations, ranging from hyperglycemic to hypoglycemic states. Both extremes can result in endothelial damage and inflammation. One study demonstrated that upregulation of Nrf2-regulated genes was inhibited in insulin-treated cerebral endothelial cells after 6 h of high glucose exposure, suggesting that insulin treatment can prevent or reduce oxidative stress (Arcambal et al., 2019). Insulin facilitates the increased expression of γ-glutamylcysteine ligase (GCLc), the rate limiting enzyme in the initial step of glutathione assembly (Langston et al., 2008). This mechanism reveals how insulin can mitigate the increased formation of ROS in the context of high glucose (González et al., 2015). The resolution of hyperglycemia with insulin treatment is also shown to decrease circulating proinflammatory cytokines TNF-α, IL6, IL-1β, and the chemokine IL-8 to or near normal control levels (Stentz et al., 2004).

The adverse effects of diabetes extend beyond the endothelium, as hyperglycemic conditions impact microglia activation, leading to increased proinflammatory cytokine production and ROS formation in both the endothelium and parenchymal microglia. Chronic hyperglycemia induces an increase in microglia frequency in the hippocampus (Wanrooy et al., 2018), indicating that excess glucose prompts their migration and proinflammatory activation. This is supported by *in vitro* evidence demonstrating that hyperglycemic conditions induce a proinflammatory phenotype in BV-2 microglia cells (Iannucci et al., 2022). Moreover, hyperinsulinemia, a phenomenon associated with insulin resistance, results in high levels of insulin and glucose in blood, leading to an increase in the release of proinflammatory cytokines, promoting microglial proliferation and proinflammatory polarization *in vivo* (Yang et al., 2022). Although many studies primarily focus on the impact of high glucose, it’s worth noting that fluctuations in glucose levels can also have significant effects. In a BV-2 *in vitro* model, shifting from a high to normal glucose concentrations kept microglia in a proinflammatory and metabolically stressed state, with increased signs of autophagy (Hsieh et al., 2019). Interestingly, an *in vivo* mouse study altered the age at which hyperglycemia was induced, resulting in varying degrees of neuroinflammation. Younger mice exposed to a prolonged high-fat diet and hyperglycemia showed increased insulin, glucose, and frequency of activated microglia in the dentate gyrus of the hippocampal formation and cornu ammonis of the hippocampus (Yao et al., 2023). Similar to the endothelial dysfunction in diabetes, the metabolic changes observed in microglia are mediated through signaling cascades involving mitogen-activated protein kinase (MPK), PI3K/Akt, and NF-κB (Hsieh et al., 2019).

## Systemic inflammation

Inflammation within the CNS is commonly associated with brain microvasculature injuries and dysfunction, as previously described. Interestingly, even infectious entities with little to no CNS penetrance can stimulate inflammatory responses within the brain parenchyma, suggesting a role for systemic inflammation in the etiopathogenesis of neuroinflammation. Despite the BBB acting as a restrictive barrier, endothelial cells participate in mediating inflammation in the brain by communicating immunological signals to perivascular MΦs and neighboring microglia, which can extend to parenchymal cells. Pro-inflammatory cytokines such as IL-6, TNF-α, and IL-1β released in response to invading pathogens, as well as inflammatory conditions, can also traverse the BBB through saturable transport mechanisms (Gutierrez et al., 1993; Banks et al., 1994a,b; Threlkeld et al., 2010). Saturable transport varies for each molecule, depending on blood concentration and endothelial cell receptors. While some molecules are taken up by endothelial cells, others are able to cross into the brain parenchyma. Notably, plasma from aged individuals can promote VCAM-1 expression on endothelial cells, subsequently activating microglia (Yousef et al., 2019). VCAM-1 activation promotes active leukocyte infiltration and is an indicator of endothelial activation.

In the healthy brain, microglia are normally restricted from blood factors. Acute injury and/or neurodegenerative disease processes may weaken BBB integrity and allow the entry of blood products into the CNS compartment and stimulate microglial activation. To explore the direct impact of blood factors on microglia stimulation, without the added influence of other cells, Mendiola et al. injected wild type mouse plasma into the corpus callosum of recipient mice brains, triggering microglia transcriptional changes in microtubule organization, oxidative phosphorylation, gene expression, protein folding, cell proliferation, chromosome organization, and cell response to stress (Mendiola et al., 2023). Interestingly, injection of plasma derived from fibrinogen alpha chain deficient (Fga)−/− mice revealed significantly downregulated genes associated with ROS, like *Hmox1*, *Cox7a2*, *Slc25a5,* and disease associated genes, *Ccl12*, *Rps8*, *Rpl35*, *Atp5e*, *PSmd2*, and *Tubb5,* as compared to wildtype injected plasma. This may suggest that the coagulation factor, fibrinogen, is a key driver of microglial activation in stroke or milder hemorrhagic events in the CNS (Mendiola et al., 2023).

Circulating red blood cells (RBCs), themselves, may also stimulate BBB responses and microglial activation in the context of aging or stress. Zhang et al. demonstrate this using two-photon microscopy on male Tie2-GFP mice, expressing green fluorescent protein in endothelial cells. Animals injected with PKH26-labeled t-butyl hydroperoxide (t-BHP) treated RBCs, to induce oxidative stress, demonstrated an increase in the number of stalled or unmoving RBCs and reduced blood flow velocity in the cerebral vasculature within 1–4 h and 24-h after RBC injection, as compared to control animals injected with phosphate-buffered saline (PBS) (Zhang et al., 2023). Although increased BBB leakage was not observed, hemosiderin-iron deposits, a by-product of RBC breakdown were increased in the brain parenchyma of animals injected with stressed RBCs (Zhang et al., 2023). Microglial activation was also seen in close proximity to vessels and stalled t-BHP-treated RBCs (Zhang et al., 2023).

Injury to the BBB and CNS can result from peripheral inflammatory conditions such as organ failure and systemic inflammatory diseases, which introduce toxic metabolites into circulation that negatively impact brain health and function. For example, liver dysfunction or failure impacts the ability of the liver to filter toxic substances, such as waste products from digestion, which can result in hepatic encephalopathy. In a mouse model of hepatic encephalopathy, reduced expression of neuronal fractalkine, a molecule that promotes ‘off’ signaling to microglia, resulted in elevated microglia activation, with increased expression of CCL2, IL-6, and TNF-α (McMillin et al., 2016), which was also seen in a hepatic injury modeled by a bile duct ligation (Dhanda et al., 2018). Similarly, a model of acute pancreatitis displayed activated microglia, indicating crosstalk between the periphery and parenchymal microglial across BBB (Cabral-França et al., 2024).

Like circulating neurotoxic factors, systemic inflammation may also promote CNS injury and dysfunction. A model of persistent circulating IL-12 but without direct injury to the brain or BBB demonstrated elevated TNF-α and IFN-γ within the circulation and brain (Gaviglio et al., 2022). This was associated with microglial MHC-II upregulation and increased brain CCR2 expression, promoting recruitment of monocytes and CD8+ T cells from the peripheral blood into the CNS compartment (Gaviglio et al., 2022). additionally, a rat model of ligature-induced periodontitis was shown to induce systemic inflammation and microglial activation, with inflammatory factors, IL-1β, IL-6, IL-8, and IL-21, elevated in the peripheral blood and brain (Hu et al., 2021). Isolating the effect of specific blood factors on microglia activity is difficult and likely complex. Importantly, potential contributions of endothelial cells on neuroinflammation were not included in a number of these studies (McMillin et al., 2016; Dhanda et al., 2018; Hu et al., 2021; Cabral-França et al., 2024), which may reveal key insight into the mechanistic pathways of neuroinflammatory activation from the periphery. Importantly, further investigation to identify early, preclinical CNS involvement during peripheral organ injury and/or failure and systemic inflammation is needed, as this is likely to provide the greatest promise for therapeutic intervention.

Bacterial infections can occur throughout the body, and in severe inflammatory conditions, such as sepsis, acute or severe neurological disorders, described as sepsis-associated encephalopathy, may develop. Sepsis patients exhibit upregulation of inducible nitric oxide synthase (iNOS) (Zrzavy et al., 2019), which catalyzes NOS production from L-arginine, in endothelial cells, astrocytes, and microglia, suggesting a proinflammatory state. Additionally, several studies have demonstrated that systemic bacterial infection or challenges with bacterial LPS can promote pathological changes within the CNS, with increase neuronal cell death and microglial activation, resulting in cognitive decline (Cunningham et al., 2005; McManus et al., 2014; Chouhan et al., 2021). It’s worth noting that patients with sepsis exhibit activated microglia in the absence of any chronic neurodegeneration disorder (Lemstra et al., 2007; Zrzavy et al., 2019). Importantly, microglia activation is seen in both systemic bacterial infection with inflammation and without significant peripheral inflammation (Zrzavy et al., 2019). Moreover, microglia return to their “resting” morphology after systemic bacterial infection, even though the brain shows elevated proinflammatory mediators, including INF-γ, IL-1β, and IL-12 (Puntener et al., 2012), suggesting microglial inflammatory responses are not fully resolved and remain in a primed state that can augment microglial responses in the context of subsequent stimuli. This same study found that cerebral vasculature experiences prolonged up-regulation of MHCI and MHCII, following systemic bacterial infection (Puntener et al., 2012), suggesting cells of the vasculature also remain in a primed state. The inflammation of the endothelial cells raises the possibility of BBB leakiness, potentially allowing inflammatory mediators, including bacterial products to enter the brain parenchyma and further stimulate microglial immune responses.

Additional evidence of systemic inflammation effecting the CNS is seen in a mouse model of intraperitoneal LPS injection, which revealed morphological changes in microglia after 3 h, suggestive of an activated state, that persisted up to one week post injection (Yang et al., 2013). The most significant morphological changes and microglia density were seen in the substantia nigra, which also correlated with TNF-α receptor 1 expression (Yang et al., 2013). Interestingly, BBB disruption and permeability were greatest in brain regions with higher microglia density (Yang et al., 2013). In a separate mouse study, repeated LPS, given intravenously over four consecutive days, revealed BBB disruption and increased density of activated microglia (Kokona et al., 2018). Microglia persisted in an activated state 3 days after the final exposure to LPS, suggesting involvement of other factors in maintaining microglial activation (Kokona et al., 2018). While the mechanisms underlying LPS-mediated reduction in BBB integrity and microglial activation are not well defined, peripheral exposure of mice to LPS was shown to induce rapid activation of endothelial NF-kB through TLR4 stimulation. Consequently, microglia activation occurs after endothelial cell activation, which suggest the endothelial cells may mediate inflammation and BBB disruption (Kodali et al., 2021).

In the context of existing neuroinflammatory disease, infection, organ injury/failure, and/or systemic inflammation, can perpetuate further cerebral vascular and CNS injury. In mice treated with 1-methyl-4-phenyl-1,2,3,6-tetrahydropyridine (MPTP) to model PD, intraperitoneal LPS exposure increased microglia activation and decreased expression of occludin, increasing BBB permeability (García-Domínguez et al., 2018). Similarly, in a rat MCAO model, repeated doses of LPS peripheral injections over 8 h increased microglia frequency, leukocyte infiltrate, and ischemic injury that was seen 3 days after MCAO (Langdon et al., 2010). Together, these studies demonstrate that existing injury in the brain increases an individuals risk for further and/or prolonged injury with infection and other diseases that are do not directly impact the CNS.

Similar to bacteria, systemic viral infections often lead to marked expression of proinflammatory cytokines and vascular complications, such as thrombosis and/or hypoxemia (Ishiguro et al., 2019; Pajo et al., 2021; Neufeldt et al., 2022; Motta et al., 2023). A recent example of this is infection with severe acute respiratory syndrome coronavirus 2 (SARS-CoV-2) virus, the etiological agent of coronavirus disease 2019 (COVID-19). Although SARS-CoV-2 was originally hypothesized to induce neurological symptoms, such as loss of smell and taste and altered consciousness, through direct infection of the brain, multiple case reports demonstrate little to no virus within the CNS compartment, including among patients that died due to severe disease (Schurink et al., 2020; Fullard et al., 2021; Thakur et al., 2021; Lebrun et al., 2023; Song et al., 2023). Virus has been reported in brain endothelium or olfactory epithelium/mucosa in infected human subjects and non-human primates (NHPs), however, this does not extend to cells residing within the brain parenchyma (Meinhardt et al., 2021; Thakur et al., 2021; Rutkai et al., 2022). Although these findings do not conclusively rule out SARS-CoV-2 infection of the CNS, the evidence suggests that it is unlikely and neurological manifestations of infection may be largely due to systemic inflammatory responses to infection, as well as peripheral organ injury. Importantly, a study of SARS-CoV-2 infection in aged, diabetic NHPs revealed infection of neurons within the olfactory cortex and interconnected regions (Beckman et al., 2022). The translation of these findings to human disease is unclear, but suggest existing vascular injury, reduced BBB integrity, and/or neuroinflammation in the context of diabetes and advanced age, may increase an individual’s risk for CNS infection.

With limited information supporting SARS-CoV-2 infection of the brain, neuroinflammation appears to be a consequence of vascular disturbances and activation of inflammatory mediators (Constant et al., 2021; Thakur et al., 2021; Rutkai et al., 2022). The saturable transport of proinflammatory cytokines across the BBB suggest that some proinflammatory cytokines freely enter the brain parenchyma, while others activate endothelial cells (Banks et al., 1994a,b; Sadowska et al., 2015). This raises the possibility that other infections that primarily affect the periphery may not have been thoroughly examined for their neurological impact in human disease. For example, non-neurotropic mouse adapted influenza viral infections, such as CA/09 H1N1, maHK68 H7N7, and A/PR/8/34 H1N1, have been shown to increase microglia accumulation and activation (Jurgens et al., 2012; Sadasivan et al., 2015; Hosseini et al., 2018). Interestingly, this is seen in some models without evidence of leukocyte infiltration or reduced BBB integrity (Sadasivan et al., 2015).

Examining the immunomodulatory impact of pre-existing proinflammatory conditions, such as stress, on subsequent infection, rats subjected to chronic psychosocial stress followed by systemic exposure of polyriboinosinic-polyribocytidilic acid (polyl:C) to mimic infection, experienced prolonged and increased pain sensitivity, or allodynia, and depressive-like behavior, as compared to control animals, as well as enhanced microglia activation (Chijiwa et al., 2015). This suggests that existing comorbidities associated with chronic inflammation, such as aging, diabetes, or cardiovascular disease, may augment microglia responses to subsequent infection, potentially through endothelial cell activation that stimulates microglial involvement at the BBB. Furthermore, it may be crucial to consider the impact of viral infection at different developmental stages. For instance, neonatal male mice challenged with murine cytomegalovirus (MCMV) demonstrated behavioral deficits, accompanied by increased microglial phagocytic activity and loss of excitatory synapses (Schwabenland et al., 2023). This study underscores the importance of exploring the consequences of systemic infection on the establishment of neural circuitry in the developing brain, which has significant microglial involvement.

## Potential therapeutic strategies

Inflammation and heightened metabolic activity in the cells comprising the BBB play a pivotal role in exacerbating injury and damage resulting from stroke, as well as that associated with comorbid conditions that cause chronic systemic inflammation. Common factors contributing to BBB dysfunction and inflammatory activation, as discussed earlier, present potential targets for therapeutic intervention. Two noteworthy players in this arena are NF-κB and Nrf2, which mediate diverse effects on inflammatory and oxidative stress pathologies. Modulating the expression and/or activation of these molecules holds promise as viable strategies for dampening inflammation and subsequent free radical production seen in stroke, diabetes, and other inflammatory conditions.

NF-κB is a major driver of inflammation by promoting transcription of cytokines and chemokines. In stroke and chronic inflammation, HIF-1α is stabilized downstream of NF-κB activation. Recently propofol, an intravenous anesthetic with anti-neuroinflammatory properties, was shown to mitigate induced oxidative damage in BV-2 cells (Peng et al., 2020). This effect was accompanied by increased superoxide dismutase (SOD) and total antioxidant capacity. Moreover, silencing of NF-κB in stressed cells yielded downregulation of HIF-1α and IL-1β (Peng et al., 2020). In the realm of herbal medicine, panax notoginseng saponins (PNS) was explored for its anti-inflammatory effects after acute ischemic stroke. PNS was found to downregulate HIF-1α/pyruvate kinase isoform PKM2/STAT3 signaling in microglia. This intervention resulted in reduced microglia activation and lowered expression of downstream inflammatory cytokines IL-1β and TNF-α in the peri-infarction regions (Gao et al., 2022). Melatonin, a serotonin derivative known to scavenge free radicals and induce Nrf2-mediated antioxidant enzymes, also exhibits an anti-inflammatory role by inhibiting the NF-κB activating cascade (Jung et al., 2010; Negi et al., 2011).

Similarly, two root-derived herbal compounds, baicalin and glycyrrhizin, exert anti-inflammatory effects through different pathways, merging on NF-κB activation. Upon baicalin pre-treatment, LPS induced BV-2 microglia expression of NO, iNOS, IL-1β, PGE2, ROS, IL-6, TNF-α, COX-2 decreased and TLR4/NF-κB pathway activation was reduced (Yan et al., 2020; Li et al., 2022; Pan et al., 2022). Baicalin treated adult mice injected with LPS displayed decreased expression of neuroinflammatory markers, Iba1 and GFAP, NF-κB transcription factor, and pro-inflammatory factors (Shah et al., 2019, 2020). Glycyrrhizin, however, blocks phosphorylation of the pro-inflammatory mediator, high-mobility group box 1 (HMGB1), inhibiting its function (Kim et al., 2012). Evidence from experimental autoimmune encephalomyelitis mouse and status epilepticus rat models demonstrate glycyrrhizin reduces pro-inflammatory activation of microglia and HMGB1 expression in microglia (Sun et al., 2018; Luo et al., 2023). In other rodent models, neuroinflammation caused by LPS and stroke was lowered with glycyrrhizin treatment by reducing HMGB1-mediated TLR4-NF-κB activation (Kim et al., 2012; Barakat et al., 2014; Sun et al., 2018). The effect of glycyrrhizin is similar to that observed with the angiotensin receptor inhibitior, candesartan, which has been shown to ameliorate ischemia-associated neuroinflammation through inhibition of TLR2 and TLR4 signaling cascades in mice (Barakat et al., 2014). Compounds from the resin of *Dracaena cochinchinensis* and the flavonoid, tectorigenin, may also effectively reduce neuroinflammation through reduced NF-κB activation (Lim et al., 2018; Tang et al., 2019).

Although there is strong evidence for herbal medicine reducing neuroinflammation, there are limitations to their therapeutic potential. Passage across the BBB, dosage, and metabolism must be considered when evaluating the biological relevance of *in vitro* findings. Additionally, mouse experiments utilize high concentrations of herbal compounds that may not be viably translated to humans. Nonetheless, these natural compounds may have valuable therapeutic potential that should be explored further in relevant pre-clinical models.

Inflammation triggers free radical formation and oxidative stress, with increased ROS and decreased antioxidant molecules, including glutathione and/or SOD. As discussed above, STZ-induced diabetes results in lower Nrf2 levels, with increased ROS and subsequent oxidative stress. Activating Nrf2 holds promise as a therapeutic strategy for reducing oxidative stress by promoting expression of enzymes responsible for neutralizing ROS. By targeting the Nrf2 signaling pathway with compounds, such as osthole, 5-hydroxymethyl-2-furfural (5-HMF), and paeonol, researchers have effectively reduced oxidative damage and BBB permeability in mouse models of ischemia (Zhao et al., 2014; Chen et al., 2015; Ya et al., 2017). In the context of diabetes treatment, insulin is known to possess additional anti-inflammatory effects, including the activation of the Nrf2 signaling pathway, which promotes the expression of antioxidant enzymes like total SOD, catalase (CAT), and glutathione peroxidase (Song et al., 2018). In addition, much like endogenous antioxidant mechanisms, exogenous antioxidants function as free radical scavengers, neutralizing ROS. Numerous studies have explored the ability of antioxidants to reduce inflammation and oxidative damage by down-regulating NF-κB and elevating Nrf2 (Li et al., 2016; Caglayan et al., 2019; Wang et al., 2021; Barber et al., 2023; Habotta et al., 2023; Jin and Leng, 2023). Nrf2 also induces expression of brain derived neurotrophic factor (BDNF), a key molecule in synaptic plasticity and learning and memory (Tang et al., 2022). Regulation of Nrf2-BDNF activation and expression is demonstrated by *in vitro* treatment of BV-2 cells with emodin and omega-3 docosapentaenoic acid, which promote BDNF expression, potentially through elevation of Nrf2 transcription (Liu et al., 2021; Gao et al., 2022). Interestingly, BDNF levels in vasculature enriched brain lysates from STZ-induced diabetic mice was reduced (Navaratna et al., 2011), mimicking decreased levels of BDNF in hypoxia and inflammation (Wu et al., 2020; Tao et al., 2022). This suggests that Nrf2 may mediate expression of BDNF in cells comprising the vasculature and microglia to elevate the expression of antioxidant enzymes.

## Conclusion

**Figure 2:**
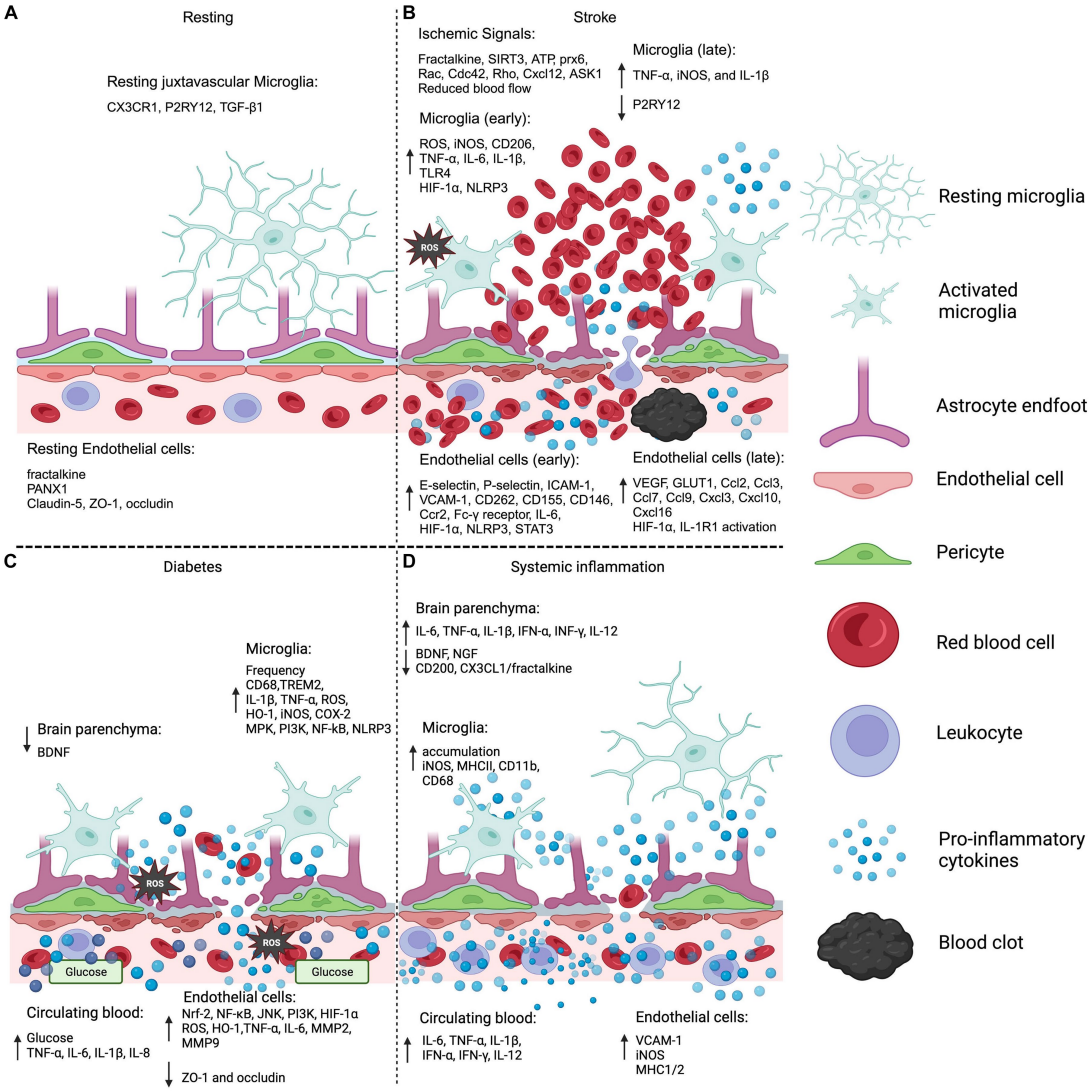
Microglia and endothelial cell BBB systemic injury. **(A)** The model displays the normal resting BBB and microglia by the vasculature. Of note, specific expression profiles of juxtavascular microglia have not been differentiated from parenchymal microglia in resting conditions. **(B)** Ischemic stroke BBB and microglia activation is shown delineating early and late expression profiles in the endothelial cells and microglia. Ischemic signals refer to the molecular signals that promote the migration of microglia to the ischemic event. **(C)** BBB and microglia inflammation and oxidative stress pathologies are demonstrated in a diabetic hyperglycemic condition. **(D)** Non-neuroinvasive systemic infection is modeled showing minimal BBB disruption with corresponding endothelial cell and microglia inflammation. Brain parenchyma refers to the information gathered from brain lysates that were not specific to one cell type. The legend displays: microglia resting and activated, light blue; astrocyte end-feet, lilac; endothelial cell, pink; pericytes, green; red blood cell, red; leukocyte, purple; pro-inflammatory cytokines, blue spheres; blood clot, black. CX3CR1, C-X3-C motif chemokine receptor 1; P2RY12, purinergic receptor P2Y12; TGF-β1, transforming growth factor beta-1; PANX1, pannexin-1; ZO-1, zonula occludens 1; SIRT3, sirtuin 3; ATP, adenosine triphosphate; prx6, peroxiredoxin 6; Cxcl12, C-X-C motif chemokine 12; ASK1, apoptosis signal-regulating kinase 1; ROS, reactive oxygen species; iNOS, inducible nitric oxide synthase; TNF-α, tumor necrosis factor-alpha; IL-6, interleukin 6; IL-1β, interleukin-1 beta; TLR4, toll-like receptor-4; HIF-1α, hypoxia inducible factor 1 subunit alpha; NLRP3, “NOD-like” receptor (NLR) family pyrin domain containing 3; ICAM-1, intercellular adhesion molecule 1; VCAM-1, vascular cell adhesion protein 1; Ccr2, C-C motif chemokine receptor 2; STAT3, signal transducer and activator of transcription 3; VEGF, vascular endothelial growth factor; GLUT1, glucose transporter 1; BDNF, brain derived neurotrophic factor; TREM2, triggering receptor expressed on myeloid cells 2; HO-1, heme oxygenase-1; COX-2, cyclooxygenase-2; MPK, mitogen activated protein kinase; PI3K, phosphoinositide 3-kinase; Nrf-2, nuclear factor erythroid 2-related factor; NF-κB, Nuclear factor kappa B; JNK, c-Jun N-terminal kinase; MMP2, matrix metalloproteinase 2; INF-γ, interferon-gamma; NGF, nerve-growth factor; CD68, cluster of differentiation 68; MHCII, major histocompatibility complex 2. Sources: Resting (Kuchler-Bopp et al., 1999; Salsman et al., 2011; Niiya et al., 2012; Sajja et al., 2014; Szalay et al., 2016; Davis et al., 2018; Xing et al., 2018), Stroke (McGeer et al., 1988; Bandera et al., 2006; Lum et al., 2007; Krady et al., 2008; Guo et al., 2009; Zhong et al., 2010; Masuda et al., 2011; Hu et al., 2012; Liu et al., 2012; Atangana et al., 2017; Cheon et al., 2017; Xing et al., 2018; Cao et al., 2019; Rashid et al., 2019; Zille et al., 2019; Hong et al., 2020; Jiang et al., 2020; Peng et al., 2020; Lubart et al., 2021; Abdi Sarabi et al., 2022; Gao et al., 2022; Pan et al., 2023; Peerlings et al., 2023; Schwabenland et al., 2023; Xu et al., 2023), Diabetes (Itoh et al., 1997; Jung et al., 2010; Secrest et al., 2013; Zhao et al., 2013; Zhang et al., 2014; Abner et al., 2016; Lively and Schlichter, 2018; Bahader et al., 2021; Misilimu et al., 2022; Liao et al., 2023), Systemic inflammation (Banks et al., 1994b; Stentz et al., 2004; Cunningham et al., 2005; Threlkeld et al., 2010; Rigato et al., 2011; McManus et al., 2014; Sajja et al., 2014; Wanrooy et al., 2018; Chouhan et al., 2021; Yang et al., 2022; Yao et al., 2023). Created with BioRender.com.

Maintaining the integrity and functionality of the BBB is essential for preserving CNS homeostasis. In this critical dynamic, microglia play a pivotal role by interacting with and communicating with the BBB. One significant aspect of this interaction is the microglial response to endothelial dysfunction within the BBB, which acts as a major contributor to inflammation and oxidative stress in the CNS. This review delves into the intricacies of these interactions, particularly in the context of stroke, diabetes, and systemic inflammation (Figure 2). It highlights a notable gap in current research: the understudied nature of microglia communication with the BBB, especially in the context of disease and/or BBB injury. Importantly, there is potential for addressing these conditions through a unified therapeutic approach, targeting the Nrf2 or NF-ĸB signaling pathways. Such interventions could be instrumental in reducing oxidative stress and inflammation, common denominators in these conditions, despite their varied etiologies and manifestations. This approach underscores the importance of considering the subtle differences in inflammation development across different diseases, while also acknowledging their consistent role in driving neuroinflammation.

Crucially, chronic neuroinflammation is a factor in the development and progression of neurodegenerative diseases and subsequent neuronal damage. Therefore, this review stresses the importance of evaluating microglia activation in scenarios of altered vascular health, elevated blood glucose levels, and indirect systemic inflammation due to pathogens. Ultimately, uncovering the mechanisms of inflammation around the BBB is key to mitigating its adverse effects. This understanding could pave the way for innovative strategies to counteract the detrimental impacts of inflammation in various neurodegenerative conditions.

## Author contributions

MM: Conceptualization, Writing – original draft, Writing – review & editing. TF: Conceptualization, Funding acquisition, Supervision, Writing – original draft, Writing – review & editing.

## References

[ref1] Abdi Sarabi M. Shiri A. Aghapour M. Reichardt C. Brandt S. Mertens P. R.. (2022). Normoxic HIF-1alpha stabilization caused by local inflammatory factors and its consequences in human coronary artery endothelial cells. Cells 11:3878. doi: 10.3390/cells11233878, PMID: 36497143 PMC9737288

[ref2] Abdul Y. Li W. Ward R. Abdelsaid M. Hafez S. Dong G.. (2021). Deferoxamine treatment prevents post-stroke Vasoregression and neurovascular unit remodeling leading to improved functional outcomes in type 2 Male diabetic rats: role of endothelial Ferroptosis. Transl. Stroke Res. 12, 615–630. doi: 10.1007/s12975-020-00844-7, PMID: 32875455 PMC7917163

[ref3] Abner E. L. Nelson P. T. Kryscio R. J. Schmitt F. A. Fardo D. W. Woltjer R. L.. (2016). Diabetes is associated with cerebrovascular but not Alzheimer's disease neuropathology. Alzheimers Dement. 12, 882–889. doi: 10.1016/j.jalz.2015.12.006, PMID: 26812281 PMC4958598

[ref4] Aggarwal A. Khera A. Singh I. Sandhir R. (2015). S-nitrosoglutathione prevents blood-brain barrier disruption associated with increased matrix metalloproteinase-9 activity in experimental diabetes. J. Neurochem. 132, 595–608. doi: 10.1111/jnc.12939, PMID: 25187090

[ref5] Ahn S. J. Anrather J. Nishimura N. Schaffer C. B. (2018). Diverse inflammatory response after cerebral microbleeds includes coordinated microglial migration and proliferation. Stroke 49, 1719–1726. doi: 10.1161/STROKEAHA.117.020461, PMID: 29844029 PMC6019563

[ref6] Akhtar N. Kamran S. Singh R. Malik R. A. Deleu D. Bourke P. J.. (2019). The impact of diabetes on outcomes after acute ischemic stroke: a prospective observational study. J. Stroke Cerebrovasc. Dis. 28, 619–626. doi: 10.1016/j.jstrokecerebrovasdis.2018.11.003, PMID: 30545720

[ref7] Alvarez J. I. Dodelet-Devillers A. Kebir H. Ifergan I. Fabre P. J. Terouz S.. (2011). The hedgehog pathway promotes blood-brain barrier integrity and CNS immune quiescence. Science 334, 1727–1731. doi: 10.1126/science.1206936, PMID: 22144466

[ref8] Arbaizar-Rovirosa M. Gallizioli M. Lozano J. J. Sidorova J. Pedragosa J. Figuerola S.. (2023). Transcriptomics and translatomics identify a robust inflammatory gene signature in brain endothelial cells after ischemic stroke. J. Neuroinflammation 20:207. doi: 10.1186/s12974-023-02888-6, PMID: 37691115 PMC10494365

[ref9] Arcambal A. Taïlé J. Rondeau P. Viranaïcken W. Meilhac O. Gonthier M. P. (2019). Hyperglycemia modulates redox, inflammatory and vasoactive markers through specific signaling pathways in cerebral endothelial cells: insights on insulin protective action. Free Radic. Biol. Med. 130, 59–70. doi: 10.1016/j.freeradbiomed.2018.10.430, PMID: 30359759

[ref10] Arnold C. N. Pirie E. Dosenovic P. McInerney G. M. Xia Y. Wang N.. (2012). A forward genetic screen reveals roles for Nfkbid, Zeb1, and Ruvbl2 in humoral immunity. Proc. Natl. Acad. Sci. U.S.A. 109, 12286–12293. doi: 10.1073/pnas.1209134109, PMID: 22761313 PMC3411946

[ref11] Atangana E. Schneider U. C. Blecharz K. Magrini S. Wagner J. Nieminen-Kelhä M.. (2017). Intravascular inflammation triggers intracerebral activated microglia and contributes to secondary brain injury after experimental subarachnoid hemorrhage (eSAH). Transl. Stroke Res. 8, 144–156. doi: 10.1007/s12975-016-0485-3, PMID: 27477569

[ref12] Bahader G. A. Nash K. M. Almarghalani D. A. Alhadidi Q. McInerney M. F. Shah Z. A. (2021). Type-I diabetes aggravates post-hemorrhagic stroke cognitive impairment by augmenting oxidative stress and neuroinflammation in mice. Neurochem. Int. 149:105151. doi: 10.1016/j.neuint.2021.105151, PMID: 34348124 PMC8387457

[ref13] Bandera E. Botteri M. Minelli C. Sutton A. Abrams K. R. Latronico N. (2006). Cerebral blood flow threshold of ischemic penumbra and infarct core in acute ischemic stroke: a systematic review. Stroke 37, 1334–1339. doi: 10.1161/01.STR.0000217418.29609.22 16574919

[ref14] Banks W. A. Kastin A. J. Ehrensing C. A. (1994b). Blood-borne interleukin-1 alpha is transported across the endothelial blood-spinal cord barrier of mice. J. Physiol. 479, 257–264. doi: 10.1113/jphysiol.1994.sp020293, PMID: 7799225 PMC1155744

[ref15] Banks W. A. Kastin A. J. Gutierrez E. G. (1994a). Penetration of interleukin-6 across the murine blood-brain barrier. Neurosci. Lett. 179, 53–56. doi: 10.1016/0304-3940(94)90933-4, PMID: 7845624

[ref16] Barakat W. Safwet N. El-Maraghy N. N. Zakaria M. N. (2014). Candesartan and glycyrrhizin ameliorate ischemic brain damage through downregulation of the TLR signaling cascade. Eur. J. Pharmacol. 724, 43–50. doi: 10.1016/j.ejphar.2013.12.032, PMID: 24378346

[ref17] Barber K. Mendonca P. Evans J. A. Soliman K. F. A. (2023). Antioxidant and anti-inflammatory mechanisms of Cardamonin through Nrf2 activation and NF-ĸB suppression in LPS-activated BV-2 microglial cells. Int. J. Mol. Sci. 24:10872. doi: 10.3390/ijms241310872, PMID: 37446045 PMC10341801

[ref18] Beckman D. Bonillas A. Diniz G. B. Ott S. Roh J. W. Elizaldi S. R.. (2022). SARS-CoV-2 infects neurons and induces neuroinflammation in a non-human primate model of COVID-19. Cell Rep. 41:111573. doi: 10.1016/j.celrep.2022.111573, PMID: 36288725 PMC9554328

[ref19] Bell K. F. Fowler J. H. Al-Mubarak B. Horsburgh K. Hardingham G. E. (2011). Activation of Nrf2-regulated glutathione pathway genes by ischemic preconditioning. Oxidative Med. Cell. Longev. 2011:689524, 1–7. doi: 10.1155/2011/689524 PMC3166574 21904646

[ref20] Bellut M. Papp L. Bieber M. Kraft P. Stoll G. Schuhmann M. K. (2021). NLPR3 inflammasome inhibition alleviates hypoxic endothelial cell death in vitro and protects blood-brain barrier integrity in murine stroke. Cell Death Dis. 13:20. doi: 10.1038/s41419-021-04379-z, PMID: 34930895 PMC8688414

[ref21] Bernier L. P. Bohlen C. J. York E. M. Choi H. B. Kamyabi A. Dissing-Olesen L.. (2019). Nanoscale surveillance of the brain by microglia via cAMP-regulated Filopodia. Cell Rep. 27, 2895–2908.e4. doi: 10.1016/j.celrep.2019.05.010, PMID: 31167136

[ref22] Bisht K. Okojie K. A. Sharma K. Lentferink D. H. Sun Y. Y. Chen H. R.. (2021). Capillary-associated microglia regulate vascular structure and function through PANX1-P2RY12 coupling in mice. Nat. Commun. 12:5289. doi: 10.1038/s41467-021-25590-8, PMID: 34489419 PMC8421455

[ref23] Boado R. J. Wang L. Pardridge W. M. (1994). Enhanced expression of the blood-brain barrier GLUT1 glucose transporter gene by brain-derived factors. Brain Res. Mol. Brain Res. 22, 259–267. doi: 10.1016/0169-328X(94)90054-X, PMID: 8015384

[ref24] Boghozian R. Sharma S. Narayana K. Cheema M. Brown C. E. (2023). Sex and interferon gamma signaling regulate microglia migration in the adult mouse cortex in vivo. Proc. Natl. Acad. Sci. USA 120:e2302892120. doi: 10.1073/pnas.2302892120, PMID: 37428916 PMC10629543

[ref25] Cabral-França T. Cruz F. F. Silva P. C. Pannain V. L. N. Fernandes A. Eulálio J. M. R.. (2024). Hippocampal microglia activation induced by acute pancreatic injury in rats. Dig. Dis. Sci. 69, 148–160. doi: 10.1007/s10620-023-08167-x, PMID: 37957410

[ref26] Caglayan B. Kilic E. Dalay A. Altunay S. Tuzcu M. Erten F.. (2019). Allyl isothiocyanate attenuates oxidative stress and inflammation by modulating Nrf2/HO-1 and NF-kappaB pathways in traumatic brain injury in mice. Mol. Biol. Rep. 46, 241–250. doi: 10.1007/s11033-018-4465-4, PMID: 30406889

[ref27] Cagnin A. Brooks D. J. Kennedy A. M. Gunn R. N. Myers R. Turkheimer F. E.. (2001a). *In-vivo* measurement of activated microglia in dementia. Lancet 358, 461–467. doi: 10.1016/S0140-6736(01)05625-2 11513911

[ref28] Cagnin A. Myers R. Gunn R. N. Lawrence A. D. Stevens T. Kreutzberg G. W.. (2001b). *In vivo* visualization of activated glia by [11C] (R)-PK11195-PET following herpes encephalitis reveals projected neuronal damage beyond the primary focal lesion. Brain 124, 2014–2027. doi: 10.1093/brain/124.10.2014 11571219

[ref29] Campbell D. J. Kladis A. Zhang Y. Jenkins A. J. Prior D. L. Yii M.. (2010). Increased tissue kallikrein levels in type 2 diabetes. Diabetologia 53, 779–785. doi: 10.1007/s00125-009-1645-8, PMID: 20225398

[ref30] Cao R. Li S. Yin J. Guo L. Shi J. (2019). Sirtuin 3 promotes microglia migration by upregulating CX3CR1. Cell Adhes. Migr. 13, 229–235. doi: 10.1080/19336918.2019.1629224, PMID: 31208274 PMC6601551

[ref31] Cardona A. E. Pioro E. P. Sasse M. E. Kostenko V. Cardona S. M. Dijkstra I. M.. (2006). Control of microglial neurotoxicity by the fractalkine receptor. Nat. Neurosci. 9, 917–924. doi: 10.1038/nn1715, PMID: 16732273

[ref32] Chehade J. M. Haas M. J. Mooradian A. D. (2002). Diabetes-related changes in rat cerebral occludin and zonula occludens-1 (ZO-1) expression. Neurochem. Res. 27, 249–252. doi: 10.1023/A:1014892706696 11958524

[ref33] Chen A. Q. Fang Z. Chen X. L. Yang S. Zhou Y. F. Mao L.. (2019). Microglia-derived TNF-alpha mediates endothelial necroptosis aggravating blood brain-barrier disruption after ischemic stroke. Cell Death Dis. 10:487. doi: 10.1038/s41419-019-1716-9, PMID: 31221990 PMC6586814

[ref34] Chen Z. Mao X. X. Liu A. M. Gao X. Y. Chen X. H. Ye M. Z.. (2015). Osthole, a natural coumarin improves cognitive impairments and BBB dysfunction after transient global brain ischemia in C57 BL/6J mice: involvement of Nrf2 pathway. Neurochem. Res. 40, 186–194. doi: 10.1007/s11064-014-1483-z, PMID: 25424966

[ref35] Chen S. Sun Y. Li F. Zhang X. Hu X. Zhao X.. (2022). Modulation of alpha7nAchR by melatonin alleviates ischemia and reperfusion-compromised integrity of blood-brain barrier through inhibiting HMGB1-mediated microglia activation and CRTC1-mediated neuronal loss. Cell. Mol. Neurobiol. 42, 2407–2422. doi: 10.1007/s10571-021-01122-2, PMID: 34196879 PMC11421614

[ref36] Chen B. Xie C. Shi T. Yue S. Li W. Huang G.. (2023). Activation of Swell1 in microglia suppresses neuroinflammation and reduces brain damage in ischemic stroke. Neurobiol. Dis. 176:105936. doi: 10.1016/j.nbd.2022.105936, PMID: 36511337

[ref37] Cheng G. Huang C. Deng H. Wang H. (2012). Diabetes as a risk factor for dementia and mild cognitive impairment: a meta-analysis of longitudinal studies. Intern. Med. J. 42, 484–491. doi: 10.1111/j.1445-5994.2012.02758.x, PMID: 22372522

[ref38] Cheon S. Y. Kim E. J. Kim J. M. Kam E. H. Ko B. W. Koo B. N. (2017). Regulation of microglia and macrophage polarization via apoptosis signal-regulating kinase 1 silencing after ischemic/hypoxic injury. Front. Mol. Neurosci. 10:261. doi: 10.3389/fnmol.2017.00261, PMID: 28855861 PMC5557792

[ref39] Chijiwa T. Oka T. Lkhagvasuren B. Yoshihara K. Sudo N. (2015). Prior chronic stress induces persistent polyI:C-induced allodynia and depressive-like behavior in rats: possible involvement of glucocorticoids and microglia. Physiol. Behav. 147, 264–273. doi: 10.1016/j.physbeh.2015.04.050, PMID: 25936823

[ref40] Chiot A. Zaïdi S. Iltis C. Ribon M. Berriat F. Schiaffino L.. (2020). Modifying macrophages at the periphery has the capacity to change microglial reactivity and to extend ALS survival. Nat. Neurosci. 23, 1339–1351. doi: 10.1038/s41593-020-00718-z, PMID: 33077946

[ref41] Choi I. Y. Lee J. C. Ju C. Hwang S. Cho G. S. Lee H. W.. (2011). A3 adenosine receptor agonist reduces brain ischemic injury and inhibits inflammatory cell migration in rats. Am. J. Pathol. 179, 2042–2052. doi: 10.1016/j.ajpath.2011.07.006, PMID: 21854743 PMC3181366

[ref42] Chouhan J. K. Puntener U. Booth S. G. Teeling J. L. (2021). Systemic inflammation accelerates changes in microglial and synaptic markers in an experimental model of chronic neurodegeneration. Front. Neurosci. 15:760721. doi: 10.3389/fnins.2021.760721 35058740 PMC8764443

[ref43] Close T. E. Cepinskas G. Omatsu T. Rose K. L. Summers K. Patterson E. K.. (2013). Diabetic ketoacidosis elicits systemic inflammation associated with cerebrovascular endothelial cell dysfunction. Microcirculation 20, 534–543. doi: 10.1111/micc.12053, PMID: 23441883

[ref44] Constant O. Barthelemy J. Bolloré K. Tuaillon E. Gosselet F. Chable-Bessia C.. (2021). SARS-CoV-2 poorly replicates in cells of the human blood-brain barrier without associated deleterious effects. Front. Immunol. 12:697329. doi: 10.3389/fimmu.2021.697329, PMID: 34386007 PMC8353323

[ref45] Császár E. Lénárt N. Cserép C. Környei Z. Fekete R. Pósfai B.. (2022). Microglia modulate blood flow, neurovascular coupling, and hypoperfusion via purinergic actions. J. Exp. Med. 219:e20211071. doi: 10.1084/jem.20211071, PMID: 35201268 PMC8932534

[ref46] Cunningham C. Wilcockson D. C. Campion S. Lunnon K. Perry V. H. (2005). Central and systemic endotoxin challenges exacerbate the local inflammatory response and increase neuronal death during chronic neurodegeneration. J. Neurosci. 25, 9275–9284. doi: 10.1523/JNEUROSCI.2614-05.2005, PMID: 16207887 PMC6725757

[ref47] Davalos D. Grutzendler J. Yang G. Kim J. V. Zuo Y. Jung S.. (2005). ATP mediates rapid microglial response to local brain injury *in vivo*. Nat. Neurosci. 8, 752–758. doi: 10.1038/nn1472, PMID: 15895084

[ref48] Davis R. L. Buck D. J. McCracken K. Cox G. W. Das S. (2018). Interleukin-1β-induced inflammatory signaling in C20 human microglial cells. Neuroimmunol. Neuroinflamm. 2018. doi: 10.20517/2347-8659.2018.60

[ref49] de Oliveira J. Engel D. F. de Paula G. C. dos Santos D. B. Lopes J. B. Farina M.. (2020). High cholesterol diet exacerbates blood-brain barrier disruption in LDLr−/− mice: impact on cognitive function. J. Alzheimers Dis. 78, 97–115. doi: 10.3233/JAD-200541, PMID: 32925052 PMC7683087

[ref50] Dhanda S. Gupta S. Halder A. Sunkaria A. Sandhir R. (2018). Systemic inflammation without gliosis mediates cognitive deficits through impaired BDNF expression in bile duct ligation model of hepatic encephalopathy. Brain Behav. Immun. 70, 214–232. doi: 10.1016/j.bbi.2018.03.002, PMID: 29518527

[ref51] Ding H. Aljofan M. Triggle C. R. (2007). Oxidative stress and increased eNOS and NADPH oxidase expression in mouse microvessel endothelial cells. J. Cell. Physiol. 212, 682–689. doi: 10.1002/jcp.21063, PMID: 17443690

[ref52] Dobi A. Rosanaly S. Devin A. Baret P. Meilhac O. Harry G. J.. (2021). Advanced glycation end-products disrupt brain microvascular endothelial cell barrier: the role of mitochondria and oxidative stress. Microvasc. Res. 133:104098. doi: 10.1016/j.mvr.2020.104098, PMID: 33075405 PMC8782206

[ref53] Dudiki T. Meller J. Mahajan G. Liu H. Zhevlakova I. Stefl S.. (2020). Microglia control vascular architecture via a TGFbeta1 dependent paracrine mechanism linked to tissue mechanics. Nat. Commun. 11:986. doi: 10.1038/s41467-020-14787-y, PMID: 32080187 PMC7033106

[ref54] Dufort C. Wang Y. Hu X. (2022). Microglia: active participants in brain capillary function. J. Cereb. Blood Flow Metab. 42, 2161–2163. doi: 10.1177/0271678X221119292, PMID: 35942567 PMC9580179

[ref55] Fanne R. A. Nassar T. Yarovoi S. Rayan A. Lamensdorf I. Karakoveski M.. (2010). Blood-brain barrier permeability and tPA-mediated neurotoxicity. Neuropharmacology 58, 972–980. doi: 10.1016/j.neuropharm.2009.12.017, PMID: 20060006 PMC4423729

[ref56] Fullard J. F. Lee H. C. Voloudakis G. Suo S. Javidfar B. Shao Z.. (2021). Single-nucleus transcriptome analysis of human brain immune response in patients with severe COVID-19. Genome Med. 13:118. doi: 10.1186/s13073-021-00933-8, PMID: 34281603 PMC8287557

[ref57] Funahashi J. Sekido R. Murai K. Kamachi Y. Kondoh H. (1993). Delta-crystallin enhancer binding protein delta EF1 is a zinc finger-homeodomain protein implicated in postgastrulation embryogenesis. Development 119, 433–446. doi: 10.1242/dev.119.2.433, PMID: 7904558

[ref58] Gao X. Bayraktutan U. (2023). TNF-alpha evokes blood-brain barrier dysfunction through activation of rho-kinase and neurokinin 1 receptor. Immunobiology 228:152706. doi: 10.1016/j.imbio.2023.152706, PMID: 37454559

[ref59] Gao L. L. Wang Z. H. Mu Y. H. Liu Z. L. Pang L. (2022). Emodin promotes autophagy and prevents apoptosis in Sepsis-associated encephalopathy through activating BDNF/TrkB signaling. Pathobiology 89, 135–145. doi: 10.1159/000520281, PMID: 34872094 PMC9227694

[ref60] Gao J. Yao M. Zhang W. Yang B. Yuan G. Liu J. X.. (2022). Panax notoginseng saponins alleviates inflammation induced by microglial activation and protects against ischemic brain injury via inhibiting HIF-1alpha/PKM2/STAT3 signaling. Biomed. Pharmacother. 155:113479. doi: 10.1016/j.biopha.2022.113479, PMID: 36271540

[ref61] García-Domínguez I. Veselá K. García-Revilla J. Carrillo-Jiménez A. Roca-Ceballos M. A. Santiago M.. (2018). Peripheral inflammation enhances microglia response and Nigral dopaminergic cell death in an in vivo MPTP model of Parkinson's disease. Front. Cell. Neurosci. 12:398. doi: 10.3389/fncel.2018.00398, PMID: 30459561 PMC6232526

[ref62] Garner K. M. Amin R. Johnson R. W. Scarlett E. J. Burton M. D. (2018). Microglia priming by interleukin-6 signaling is enhanced in aged mice. J. Neuroimmunol. 324, 90–99. doi: 10.1016/j.jneuroim.2018.09.002, PMID: 30261355 PMC6699492

[ref63] Gaviglio E. A. Peralta Ramos J. M. Arroyo D. S. Bussi C. Iribarren P. Rodriguez-Galan M. C. (2022). Systemic sterile induced-co-expression of IL-12 and IL-18 drive IFN-gamma-dependent activation of microglia and recruitment of MHC-II-expressing inflammatory monocytes into the brain. Int. Immunopharmacol. 105:108546. doi: 10.1016/j.intimp.2022.108546, PMID: 35074570 PMC8901210

[ref64] Ginhoux F. Greter M. Leboeuf M. Nandi S. See P. Gokhan S.. (2010). Fate mapping analysis reveals that adult microglia derive from primitive macrophages. Science 330, 841–845. doi: 10.1126/science.1194637, PMID: 20966214 PMC3719181

[ref65] González M. Rojas S. Avila P. Cabrera L. Villalobos R. Palma C.. (2015). Insulin reverses D-glucose-increased nitric oxide and reactive oxygen species generation in human umbilical vein endothelial cells. PLoS One 10:e0122398. doi: 10.1371/journal.pone.0122398, PMID: 25875935 PMC4397070

[ref66] Grossmann R. Stence N. Carr J. Fuller L. Waite M. Dailey M. E. (2002). Juxtavascular microglia migrate along brain microvessels following activation during early postnatal development. Glia 37, 229–240. doi: 10.1002/glia.10031, PMID: 11857681

[ref67] Guo S. Miyake M. Liu K. J. Shi H. (2009). Specific inhibition of hypoxia inducible factor 1 exaggerates cell injury induced by *in vitro* ischemia through deteriorating cellular redox environment. J. Neurochem. 108, 1309–1321. doi: 10.1111/j.1471-4159.2009.05877.x, PMID: 19183269 PMC2666308

[ref68] Gutierrez E. G. Banks W. A. Kastin A. J. (1993). Murine tumor necrosis factor alpha is transported from blood to brain in the mouse. J. Neuroimmunol. 47, 169–176. doi: 10.1016/0165-5728(93)90027-V 8370768

[ref69] Habotta O. Ateya A. Saleh R. M. El-Ashry E. S. (2023). Thiamethoxam evoked neural oxido-inflammatory stress in male rats through modulation of Nrf2/NF-kB/iNOS signaling and inflammatory cytokines: neuroprotective effect of Silymarin. Neurotoxicology 96, 28–36. doi: 10.1016/j.neuro.2023.03.004, PMID: 36958429

[ref70] Han X. Li Q. Lan X. el-Mufti L. Ren H. Wang J. (2019). Microglial depletion with Clodronate liposomes increases Proinflammatory cytokine levels, induces astrocyte activation, and damages blood vessel integrity. Mol. Neurobiol. 56, 6184–6196. doi: 10.1007/s12035-019-1502-9, PMID: 30734229 PMC6684378

[ref71] Hartmann D. A. Berthiaume A. A. Grant R. I. Harrill S. A. Koski T. Tieu T.. (2021). Brain capillary pericytes exert a substantial but slow influence on blood flow. Nat. Neurosci. 24, 633–645. doi: 10.1038/s41593-020-00793-2, PMID: 33603231 PMC8102366

[ref72] Haruwaka K. Ikegami A. Tachibana Y. Ohno N. Konishi H. Hashimoto A.. (2019). Dual microglia effects on blood brain barrier permeability induced by systemic inflammation. Nat. Commun. 10:5816. doi: 10.1038/s41467-019-13812-z, PMID: 31862977 PMC6925219

[ref73] Hawkins B. T. Lundeen T. F. Norwood K. M. Brooks H. L. Egleton R. D. (2007). Increased blood-brain barrier permeability and altered tight junctions in experimental diabetes in the rat: contribution of hyperglycaemia and matrix metalloproteinases. Diabetologia 50, 202–211. doi: 10.1007/s00125-006-0485-z, PMID: 17143608

[ref74] Hikage F. Lennikov A. Mukwaya A. Lachota M. Ida Y. Utheim T. P.. (2021). NF-kappaB activation in retinal microglia is involved in the inflammatory and neovascularization signaling in laser-induced choroidal neovascularization in mice. Exp. Cell Res. 403:112581. doi: 10.1016/j.yexcr.2021.112581, PMID: 33811906 PMC8479856

[ref75] Hoek R. M. Ruuls S. R. Murphy C. A. Wright G. J. Goddard R. Zurawski S. M.. (2000). Down-regulation of the macrophage lineage through interaction with OX2 (CD200). Science 290, 1768–1771. doi: 10.1126/science.290.5497.1768, PMID: 11099416

[ref76] Hofmann M. A. Schiekofer S. Isermann B. Kanitz M. Henkels M. Joswig M.. (1999). Peripheral blood mononuclear cells isolated from patients with diabetic nephropathy show increased activation of the oxidative-stress sensitive transcription factor NF-kappa B. Diabetologia 42, 222–232. doi: 10.1007/s001250051142, PMID: 10064103

[ref77] Hong J. Kim Y. Yanpallewar S. Lin P. C. (2020). The rho/Rac guanine nucleotide exchange factor Vav1 regulates Hif-1alpha and Glut-1 expression and glucose uptake in the brain. Int. J. Mol. Sci. 21:1341. doi: 10.3390/ijms21041341, PMID: 32079227 PMC7072975

[ref78] Hosseini S. Wilk E. Michaelsen-Preusse K. Gerhauser I. Baumgärtner W. Geffers R.. (2018). Long-term Neuroinflammation induced by influenza a virus infection and the impact on hippocampal neuron morphology and function. J. Neurosci. 38, 3060–3080. doi: 10.1523/JNEUROSCI.1740-17.2018, PMID: 29487124 PMC6596076

[ref79] Hou B. Jiang C. Wang D. Wang G. Wang Z. Zhu M.. (2020). Pharmacological targeting of CSF1R inhibits microglial proliferation and aggravates the progression of cerebral ischemic pathology. Front. Cell. Neurosci. 14:267. doi: 10.3389/fncel.2020.00267, PMID: 33177990 PMC7596178

[ref80] Hristova M. Cuthill D. Zbarsky V. Acosta-Saltos A. Wallace A. Blight K.. (2010). Activation and deactivation of periventricular white matter phagocytes during postnatal mouse development. Glia 58, 11–28. doi: 10.1002/glia.20896, PMID: 19544386

[ref81] Hsieh C. F. Liu C. K. Lee C. T. Yu L. E. Wang J. Y. (2019). Acute glucose fluctuation impacts microglial activity, leading to inflammatory activation or self-degradation. Sci. Rep. 9:840. doi: 10.1038/s41598-018-37215-0, PMID: 30696869 PMC6351546

[ref82] Hu Q. Du Q. Yu W. Dong X. (2022). 2-Methoxyestradiol alleviates Neuroinflammation and brain edema in early brain injury after subarachnoid hemorrhage in rats. Front. Cell. Neurosci. 16:869546. doi: 10.3389/fncel.2022.869546, PMID: 35558877 PMC9087802

[ref83] Hu X. Li P. Guo Y. Wang H. Leak R. K. Chen S.. (2012). Microglia/macrophage polarization dynamics reveal novel mechanism of injury expansion after focal cerebral ischemia. Stroke 43, 3063–3070. doi: 10.1161/STROKEAHA.112.659656, PMID: 22933588

[ref84] Hu Y. Zhang X. Zhang J. Xia X. Li H. Qiu C.. (2021). Activated STAT3 signaling pathway by ligature-induced periodontitis could contribute to neuroinflammation and cognitive impairment in rats. J. Neuroinflammation 18:80. doi: 10.1186/s12974-021-02071-9, PMID: 33757547 PMC7986277

[ref85] Huang M. Wan Y. Mao L. He Q. W. Xia Y. P. Li M.. (2017). Inhibiting the migration of M1 microglia at Hyperacute period could improve outcome of tMCAO rats. CNS Neurosci. Ther. 23, 222–232. doi: 10.1111/cns.12665, PMID: 27991729 PMC6492671

[ref86] Iannucci J. Rao H. V. Grammas P. (2022). High glucose and hypoxia-mediated damage to human brain microvessel endothelial cells induces an altered, pro-inflammatory phenotype in BV-2 microglia in vitro. Cell. Mol. Neurobiol. 42, 985–996. doi: 10.1007/s10571-020-00987-z, PMID: 33136275 PMC8942976

[ref87] Igarashi Y. Utsumi H. Chiba H. Yamada-Sasamori Y. Tobioka H. Kamimura Y.. (1999). Glial cell line-derived neurotrophic factor induces barrier function of endothelial cells forming the blood-brain barrier. Biochem. Biophys. Res. Commun. 261, 108–112. doi: 10.1006/bbrc.1999.0992, PMID: 10405331

[ref88] Ishiguro T. Matsuo K. Fujii S. Takayanagi N. (2019). Acute thrombotic vascular events complicating influenza-associated pneumonia. Respir. Med. Case. Rep. 28:100884. doi: 10.1016/j.rmcr.2019.100884, PMID: 31245274 PMC6582236

[ref89] Itoh K. Chiba T. Takahashi S. Ishii T. Igarashi K. Katoh Y.. (1997). An Nrf2/small Maf heterodimer mediates the induction of phase II detoxifying enzyme genes through antioxidant response elements. Biochem. Biophys. Res. Commun. 236, 313–322. doi: 10.1006/bbrc.1997.6943, PMID: 9240432

[ref90] Jackson L. Dumanli S. Johnson M. H. Fagan S. C. Ergul A. (2020). Microglia knockdown reduces inflammation and preserves cognition in diabetic animals after experimental stroke. J. Neuroinflammation 17:137. doi: 10.1186/s12974-020-01815-3, PMID: 32345303 PMC7189436

[ref91] Jackson-Cowan L. Eldahshan W. Dumanli S. Dong G. Jamil S. Abdul Y.. (2021). Delayed Administration of Angiotensin Receptor (AT2R) agonist C21 improves survival and preserves sensorimotor outcomes in female diabetic rats post-stroke through modulation of microglial activation. Int. J. Mol. Sci. 22:1356. doi: 10.3390/ijms22031356, PMID: 33572986 PMC7866408

[ref92] Jiang Q. Geng X. Warren J. Eugene Paul Cosky E. Kaura S. Stone C.. (2020). Hypoxia inducible factor-1alpha (HIF-1alpha) mediates NLRP3 Inflammasome-dependent-Pyroptotic and apoptotic cell death following ischemic stroke. Neuroscience 448, 126–139. doi: 10.1016/j.neuroscience.2020.09.036, PMID: 32976985

[ref93] Jin T. Leng B. (2023). Cynaropicrin averts the oxidative stress and Neuroinflammation in ischemic/reperfusion injury through the modulation of NF-kB. Appl. Biochem. Biotechnol. 195, 5424–5438. doi: 10.1007/s12010-022-04060-x, PMID: 35838888 PMC10457408

[ref94] Johnson L. A. Jackson D. G. (2010). Inflammation-induced secretion of CCL21 in lymphatic endothelium is a key regulator of integrin-mediated dendritic cell transmigration. Int. Immunol. 22, 839–849. doi: 10.1093/intimm/dxq435, PMID: 20739459

[ref95] Jung K. H. Hong S. W. Zheng H. M. Lee H. S. Lee H. Lee D. H.. (2010). Melatonin ameliorates cerulein-induced pancreatitis by the modulation of nuclear erythroid 2-related factor 2 and nuclear factor-kappaB in rats. J. Pineal Res. 48, 239–250. doi: 10.1111/j.1600-079X.2010.00748.x, PMID: 20210857

[ref96] Jurgens H. A. Amancherla K. Johnson R. W. (2012). Influenza infection induces neuroinflammation, alters hippocampal neuron morphology, and impairs cognition in adult mice. J. Neurosci. 32, 3958–3968. doi: 10.1523/JNEUROSCI.6389-11.2012, PMID: 22442063 PMC3353809

[ref97] Kacem K. Lacombe P. Seylaz J. Bonvento G. (1998). Structural organization of the perivascular astrocyte endfeet and their relationship with the endothelial glucose transporter: a confocal microscopy study. Glia 23, 1–10. doi: 10.1002/(SICI)1098-1136(199805)23:1<1::AID-GLIA1>3.0.CO;2-B, PMID: 9562180

[ref98] Kacimi R. Giffard R. G. Yenari M. A. (2011). Endotoxin-activated microglia injure brain derived endothelial cells via NF-κB, JAK-STAT and JNK stress kinase pathways. J. Inflamm. 8:7. doi: 10.1186/1476-9255-8-7, PMID: 21385378 PMC3061894

[ref99] Kayano R. Morofuji Y. Nakagawa S. Fukuda S. Watanabe D. Ozawa H.. (2018). In vitro analysis of drugs that improve hyperglycemia-induced blood-brain barrier dysfunction. Biochem. Biophys. Res. Commun. 503, 1885–1890. doi: 10.1016/j.bbrc.2018.07.131, PMID: 30060956

[ref100] Kim E. H. Kim E. S. Shin D. Kim D. Choi S. Shin Y. J.. (2021). Carnosine protects against cerebral ischemic injury by inhibiting matrix-metalloproteinases. Int. J. Mol. Sci. 22:7495. doi: 10.3390/ijms22147495 34299128 PMC8306548

[ref101] Kim S-W. Jin Y. Shin J-H. Kim I-D. Lee H-K. Park S.. (2012). Glycyrrhizic acid affords robust neuroprotection in the postischemic brain via anti-inflammatory effect by inhibiting HMGB1 phosphorylation and secretion. Neurobiol. Dis. 46, 147–156. doi: 10.1016/j.nbd.2011.12.056 22266336

[ref102] Knottnerus I. L. Ten Cate H. Lodder J. Kessels F. van Oostenbrugge R. J. (2009). Endothelial dysfunction in lacunar stroke: a systematic review. Cerebrovasc. Dis. 27, 519–526. doi: 10.1159/000212672, PMID: 19372654

[ref103] Kodali M. C. Chen H. Liao F. F. (2021). Temporal unsnarling of brain's acute neuroinflammatory transcriptional profiles reveals panendothelitis as the earliest event preceding microgliosis. Mol. Psychiatry 26, 3905–3919. doi: 10.1038/s41380-020-00955-5, PMID: 33293688 PMC7722246

[ref104] Kokona D. Ebneter A. Escher P. Zinkernagel M. S. (2018). Colony-stimulating factor 1 receptor inhibition prevents disruption of the blood-retina barrier during chronic inflammation. J. Neuroinflammation 15:340. doi: 10.1186/s12974-018-1373-4, PMID: 30541565 PMC6292111

[ref105] Krady J. K. Lin H. W. Liberto C. M. Basu A. Kremlev S. G. Levison S. W. (2008). Ciliary neurotrophic factor and interleukin-6 differentially activate microglia. J. Neurosci. Res. 86, 1538–1547. doi: 10.1002/jnr.21620, PMID: 18214991

[ref106] Krasnow S. M. Knoll J. G. Verghese S. C. Levasseur P. R. Marks D. L. (2017). Amplification and propagation of interleukin-1beta signaling by murine brain endothelial and glial cells. J. Neuroinflammation 14:133. doi: 10.1186/s12974-017-0908-4, PMID: 28668091 PMC5494131

[ref107] Kuang X. Wang L. F. Yu L. Li Y. J. Wang Y. N. He Q.. (2014). Ligustilide ameliorates neuroinflammation and brain injury in focal cerebral ischemia/reperfusion rats: involvement of inhibition of TLR4/peroxiredoxin 6 signaling. Free Radic. Biol. Med. 71, 165–175. doi: 10.1016/j.freeradbiomed.2014.03.028, PMID: 24681253

[ref108] Kuchler-Bopp S. Delaunoy J. P. Artault J. C. Zaepfel M. Dietrich J. B. (1999). Astrocytes induce several blood-brain barrier properties in non-neural endothelial cells. Neuroreport 10, 1347–1353. doi: 10.1097/00001756-199904260-00035, PMID: 10363951

[ref109] Kuno R. Wang J. Kawanokuchi J. Takeuchi H. Mizuno T. Suzumura A. (2005). Autocrine activation of microglia by tumor necrosis factor-alpha. J. Neuroimmunol. 162, 89–96. doi: 10.1016/j.jneuroim.2005.01.015 15833363

[ref110] Kuo P. C. Weng W. T. Scofield B. A. Paraiso H. C. Bojrab P. Kimes B.. (2023). Interferon-beta modulates microglial polarization to ameliorate delayed tPA-exacerbated brain injury in ischemic stroke. Front. Immunol. 14:1148069. doi: 10.3389/fimmu.2023.1148069, PMID: 37063896 PMC10104603

[ref111] Laflamme N. Lacroix S. Rivest S. (1999). An essential role of interleukin-1beta in mediating NF-kappa B activity and COX-2 transcription in cells of the blood-brain barrier in response to a systemic and localized inflammation but not during endotoxemia. J. Neurosci. 19, 10923–10930. doi: 10.1523/JNEUROSCI.19-24-10923.1999, PMID: 10594073 PMC6784955

[ref112] Langdon K. D. Maclellan C. L. Corbett D. (2010). Prolonged, 24-h delayed peripheral inflammation increases short- and long-term functional impairment and histopathological damage after focal ischemia in the rat. J. Cereb. Blood Flow Metab. 30, 1450–1459. doi: 10.1038/jcbfm.2010.23, PMID: 20332799 PMC2949250

[ref113] Langston W. Circu M. L. Aw T. Y. (2008). Insulin stimulation of gamma-glutamylcysteine ligase catalytic subunit expression increases endothelial GSH during oxidative stress: influence of low glucose. Free Radic. Biol. Med. 45, 1591–1599. doi: 10.1016/j.freeradbiomed.2008.09.013, PMID: 18926903 PMC2631205

[ref114] Lau L. H. Lew J. Borschmann K. Thijs V. Ekinci E. I. (2019). Prevalence of diabetes and its effects on stroke outcomes: a meta-analysis and literature review. J. Diab. Invest. 10, 780–792. doi: 10.1111/jdi.12932, PMID: 30220102 PMC6497593

[ref115] Lebrun L. Absil L. Remmelink M. de Mendonça R. D’Haene N. Gaspard N.. (2023). SARS-Cov-2 infection and neuropathological findings: a report of 18 cases and review of the literature. Acta Neuropathol. Commun. 11:78. doi: 10.1186/s40478-023-01566-1, PMID: 37165453 PMC10170054

[ref116] Lee J. M. Calkins M. J. Chan K. Kan Y. W. Johnson J. A. (2003). Identification of the NF-E2-related factor-2-dependent genes conferring protection against oxidative stress in primary cortical astrocytes using oligonucleotide microarray analysis. J. Biol. Chem. 278, 12029–12038. doi: 10.1074/jbc.M211558200, PMID: 12556532

[ref117] Lehrmann E. Christensen T. Zimmer J. Diemer N. H. Finsen B. (1997). Microglial and macrophage reactions mark progressive changes and define the penumbra in the rat neocortex and striatum after transient middle cerebral artery occlusion. J. Comp. Neurol. 386, 461–476. doi: 10.1002/(SICI)1096-9861(19970929)386:3<461::AID-CNE9>3.0.CO;2-#, PMID: 9303429

[ref118] Lemstra A. W. Groen in't Woud J. C. M. Hoozemans J. J. M. van Haastert E. S. Rozemuller A. J. M. Eikelenboom P.. (2007). Microglia activation in sepsis: a case-control study. J. Neuroinflammation 4:4. doi: 10.1186/1742-2094-4-4, PMID: 17224051 PMC1783646

[ref119] Li D. Lang W. Zhou C. Wu C. Zhang F. Liu Q.. (2018). Upregulation of microglial ZEB1 ameliorates brain damage after acute ischemic stroke. Cell Rep. 22, 3574–3586. doi: 10.1016/j.celrep.2018.03.011, PMID: 29590624

[ref120] Li W. Maloney R. E. Aw T. Y. (2015). High glucose, glucose fluctuation and carbonyl stress enhance brain microvascular endothelial barrier dysfunction: implications for diabetic cerebral microvasculature. Redox Biol. 5, 80–90. doi: 10.1016/j.redox.2015.03.005, PMID: 25867911 PMC4398791

[ref121] Li W. Suwanwela N. C. Patumraj S. (2016). Curcumin by down-regulating NF-kB and elevating Nrf2, reduces brain edema and neurological dysfunction after cerebral I/R. Microvasc. Res. 106, 117–127. doi: 10.1016/j.mvr.2015.12.008, PMID: 26686249

[ref122] Li B. Wang M. Chen S. Li M. Zeng J. Wu S.. (2022). Baicalin mitigates the Neuroinflammation through the TLR4/MyD88/NF-kappaB and MAPK pathways in LPS-stimulated BV-2 microglia. Biomed. Res. Int. 2022, 1–15. doi: 10.1155/2022/3263446 PMC9668451 36408278

[ref123] Li T. Zhao J. Gao H. (2022). Depletion of Arg1-positive microglia/macrophages exacerbates cerebral ischemic damage by facilitating the inflammatory response. Int. J. Mol. Sci. 23:13055. doi: 10.3390/ijms232113055 36361836 PMC9655877

[ref124] Li T. Zhao J. Xie W. Yuan W. Guo J. Pang S.. (2021). Specific depletion of resident microglia in the early stage of stroke reduces cerebral ischemic damage. J. Neuroinflammation 18:81. doi: 10.1186/s12974-021-02127-w, PMID: 33757565 PMC7986495

[ref125] Liao Y. C. Wang J. W. Guo C. Bai M. Ran Z. Wen L. M.. (2023). Cistanche tubulosa alleviates ischemic stroke-induced blood-brain barrier damage by modulating microglia-mediated neuroinflammation. J. Ethnopharmacol. 309:116269. doi: 10.1016/j.jep.2023.116269, PMID: 36863639

[ref126] Lien C. F. Mohanta S. K. Frontczak-Baniewicz M. Swinny J. D. Zablocka B. Górecki D. C. (2012). Absence of glial alpha-dystrobrevin causes abnormalities of the blood-brain barrier and progressive brain edema. J. Biol. Chem. 287, 41374–41385. doi: 10.1074/jbc.M112.400044, PMID: 23043099 PMC3510835

[ref127] Lim H. S. Kim Y. J. Kim B. Y. Park G. Jeong S. J. (2018). The anti-neuroinflammatory activity of tectorigenin pretreatment via downregulated NF-kappaB and ERK/JNK pathways in BV-2 microglial and microglia inactivation in mice with lipopolysaccharide. Front. Pharmacol. 9:462. doi: 10.3389/fphar.2018.00462, PMID: 29867470 PMC5954245

[ref128] Lin L. Wang Q. Qian K. Cao Z. Xiao J. Wang X.. (2018). bFGF protects against oxygen glucose deprivation/Reoxygenation-induced endothelial monolayer permeability via S1PR1-dependent mechanisms. Mol. Neurobiol. 55, 3131–3142. doi: 10.1007/s12035-017-0544-0, PMID: 28466272

[ref129] Liu L. F. Hu Y. Liu Y. N. Shi D. W. Liu C. da X.. (2022). Reactive oxygen species contribute to delirium-like behavior by activating CypA/MMP9 signaling and inducing blood-brain barrier impairment in aged mice following anesthesia and surgery. Front. Aging Neurosci. 14:1021129. doi: 10.3389/fnagi.2022.1021129, PMID: 36337710 PMC9629746

[ref130] Liu J. Jin X. Liu K. J. Liu W. (2012). Matrix metalloproteinase-2-mediated occludin degradation and caveolin-1-mediated claudin-5 redistribution contribute to blood-brain barrier damage in early ischemic stroke stage. J. Neurosci. 32, 3044–3057. doi: 10.1523/JNEUROSCI.6409-11.2012, PMID: 22378877 PMC3339570

[ref131] Liu B. Zhang Y. Yang Z. Liu M. Zhang C. Zhao Y.. (2021). Omega-3 DPA protected neurons from Neuroinflammation by balancing microglia M1/M2 polarizations through inhibiting NF-kappaB/MAPK p38 signaling and activating neuron-BDNF-PI3K/AKT pathways. Mar. Drugs 19:587. doi: 10.3390/md19110587, PMID: 34822458 PMC8619469

[ref132] Lively S. Schlichter L. C. (2018). Microglia responses to pro-inflammatory stimuli (LPS, IFNgamma+TNFalpha) and reprogramming by resolving cytokines (IL-4, IL-10). Front. Cell. Neurosci. 12:215. doi: 10.3389/fncel.2018.00215, PMID: 30087595 PMC6066613

[ref133] Lubart A. Benbenishty A. Har-Gil H. Laufer H. Gdalyahu A. Assaf Y.. (2021). Single cortical microinfarcts Lead to widespread microglia/macrophage migration along the white matter. Cereb. Cortex 31, 248–266. doi: 10.1093/cercor/bhaa223, PMID: 32954425

[ref134] Luchsinger J. A. Tang M. X. Stern Y. Shea S. Mayeux R. (2001). Diabetes mellitus and risk of Alzheimer's disease and dementia with stroke in a multiethnic cohort. Am. J. Epidemiol. 154, 635–641. doi: 10.1093/aje/154.7.635, PMID: 11581097

[ref135] Lum J. J. Bui T. Gruber M. Gordan J. D. DeBerardinis R. J. Covello K. L.. (2007). The transcription factor HIF-1alpha plays a critical role in the growth factor-dependent regulation of both aerobic and anaerobic glycolysis. Genes Dev. 21, 1037–1049. doi: 10.1101/gad.1529107, PMID: 17437992 PMC1855230

[ref136] Luo X. Wu J. Wu G. (2021). PPARgamma activation suppresses the expression of MMP9 by downregulating NF-kappaB post intracerebral hemorrhage. Neurosci. Lett. 752:135770. doi: 10.1016/j.neulet.2021.135770, PMID: 33636289

[ref137] Luo Z. Xu M. Zhang L. Zhang H. Xu Z. Xu Z. (2023). Glycyrrhizin regulates the HMGB1/P38MAPK signalling pathway in status epilepticus. Mol. Med. Rep. 27:45. doi: 10.3892/mmr.2023.12932, PMID: 36633134 PMC9887508

[ref138] Mäe M. A. Li T. Bertuzzi G. Raschperger E. Vanlandewijck M. He L.. (2018). Prolonged systemic hyperglycemia does not cause pericyte loss and permeability at the mouse blood-brain barrier. Sci. Rep. 8:17462. doi: 10.1038/s41598-018-35576-0, PMID: 30498224 PMC6265246

[ref139] Manley G. T. Fujimura M. Ma T. Noshita N. Filiz F. Bollen A. W.. (2000). Aquaporin-4 deletion in mice reduces brain edema after acute water intoxication and ischemic stroke. Nat. Med. 6, 159–163. doi: 10.1038/72256, PMID: 10655103

[ref140] Masuda T. Croom D. Hida H. Kirov S. A. (2011). Capillary blood flow around microglial somata determines dynamics of microglial processes in ischemic conditions. Glia 59, 1744–1753. doi: 10.1002/glia.21220, PMID: 21800362 PMC3174346

[ref141] McCaffrey G. Staatz W. D. Quigley C. A. Nametz N. Seelbach M. J. Campos C. R.. (2007). Tight junctions contain oligomeric protein assembly critical for maintaining blood-brain barrier integrity in vivo. J. Neurochem. 103, 2540–2555. doi: 10.1111/j.1471-4159.2007.04943.x, PMID: 17931362

[ref142] McGeer P. L. Itagaki S. Boyes B. E. McGeer E. G. (1988). Reactive microglia are positive for HLA-DR in the substantia nigra of Parkinson's and Alzheimer's disease brains. Neurology 38, 1285–1291. doi: 10.1212/WNL.38.8.1285, PMID: 3399080

[ref143] McGeer P. L. Itagaki S. Tago H. McGeer E. G. (1987). Reactive microglia in patients with senile dementia of the Alzheimer type are positive for the histocompatibility glycoprotein HLA-DR. Neurosci. Lett. 79, 195–200. doi: 10.1016/0304-3940(87)90696-3, PMID: 3670729

[ref144] McMahon M. Thomas N. Itoh K. Yamamoto M. Hayes J. D. (2006). Dimerization of substrate adaptors can facilitate cullin-mediated ubiquitylation of proteins by a "tethering" mechanism: a two-site interaction model for the Nrf2-Keap 1 complex. J. Biol. Chem. 281, 24756–24768. doi: 10.1074/jbc.M601119200, PMID: 16790436

[ref145] McManus R. M. Higgins S. C. Mills K. H. Lynch M. A. (2014). Respiratory infection promotes T cell infiltration and amyloid-beta deposition in APP/PS1 mice. Neurobiol. Aging 35, 109–121. doi: 10.1016/j.neurobiolaging.2013.07.025, PMID: 23993702

[ref146] McMillin M. Grant S. Frampton G. Andry S. Brown A. DeMorrow S. (2016). Fractalkine suppression during hepatic encephalopathy promotes neuroinflammation in mice. J. Neuroinflammation 13:198. doi: 10.1186/s12974-016-0674-8, PMID: 27561705 PMC5000400

[ref147] Mehrabadi A. R. Korolainen M. A. Odero G. Miller D. W. Kauppinen T. M. (2017). Poly (ADP-ribose) polymerase-1 regulates microglia mediated decrease of endothelial tight junction integrity. Neurochem. Int. 108, 266–271. doi: 10.1016/j.neuint.2017.04.014, PMID: 28461173

[ref148] Meinhardt J. Radke J. Dittmayer C. Franz J. Thomas C. Mothes R.. (2021). Olfactory transmucosal SARS-CoV-2 invasion as a port of central nervous system entry in individuals with COVID-19. Nat. Neurosci. 24, 168–175. doi: 10.1038/s41593-020-00758-5, PMID: 33257876

[ref149] Mendiola A. S. Yan Z. Dixit K. Johnson J. R. Bouhaddou M. Meyer-Franke A.. (2023). Defining blood-induced microglia functions in neurodegeneration through multiomic profiling. Nat. Immunol. 24, 1173–1187. doi: 10.1038/s41590-023-01522-0, PMID: 37291385 PMC10307624

[ref150] Mills S. A. Jobling A. I. Dixon M. A. Bui B. V. Vessey K. A. Phipps J. A.. (2021). Fractalkine-induced microglial vasoregulation occurs within the retina and is altered early in diabetic retinopathy. Proc. Natl. Acad. Sci. U. S.A. 118:e2112561118. doi: 10.1073/pnas.2112561118 34903661 PMC8713803

[ref151] Misilimu D. Li W. Chen D. Wei P. Huang Y. Li S.. (2022). Intranasal Salvinorin a improves Long-term neurological function via immunomodulation in a mouse ischemic stroke model. J. Neuroimmune Pharmacol. 17, 350–366. doi: 10.1007/s11481-021-10025-4, PMID: 34596819 PMC9726789

[ref152] Mondo E. Becker S. C. Kautzman A. G. Schifferer M. Baer C. E. Chen J.. (2020). A developmental analysis of Juxtavascular microglia dynamics and interactions with the vasculature. J. Neurosci. 40, 6503–6521. doi: 10.1523/JNEUROSCI.3006-19.2020, PMID: 32661024 PMC7486666

[ref153] Monier A. Evrard P. Gressens P. Verney C. (2006). Distribution and differentiation of microglia in the human encephalon during the first two trimesters of gestation. J. Comp. Neurol. 499, 565–582. doi: 10.1002/cne.21123, PMID: 17029271

[ref154] Monif M. Reid C. A. Powell K. L. Drummond K. J. O’Brien T. J. Williams D. A. (2016). Interleukin-1beta has trophic effects in microglia and its release is mediated by P2X7R pore. J. Neuroinflammation 13:173. doi: 10.1186/s12974-016-0621-8, PMID: 27364756 PMC4929731

[ref155] Motta C. S. Torices S. da Rosa B. G. Marcos A. C. Alvarez-Rosa L. Siqueira M.. (2023). Human brain microvascular endothelial cells exposure to SARS-CoV-2 leads to inflammatory activation through NF-kappaB non-canonical pathway and mitochondrial remodeling. Viruses 15:745. doi: 10.3390/v15030745, PMID: 36992454 PMC10056985

[ref156] Mu D. Jiang X. Sheldon R. A. Fox C. K. Hamrick S. E. G. Vexler Z. S.. (2003). Regulation of hypoxia-inducible factor 1alpha and induction of vascular endothelial growth factor in a rat neonatal stroke model. Neurobiol. Dis. 14, 524–534. doi: 10.1016/j.nbd.2003.08.020, PMID: 14678768

[ref157] Nagyőszi P. Wilhelm I. Farkas A. E. Fazakas C. Dung N. T. K. Haskó J.. (2010). Expression and regulation of toll-like receptors in cerebral endothelial cells. Neurochem. Int. 57, 556–564. doi: 10.1016/j.neuint.2010.07.002 20637248

[ref158] Navaratna D. Guo S. Z. Hayakawa K. Wang X. Gerhardinger C. Lo E. H. (2011). Decreased cerebrovascular brain-derived neurotrophic factor-mediated neuroprotection in the diabetic brain. Diabetes 60, 1789–1796. doi: 10.2337/db10-1371, PMID: 21562076 PMC3114398

[ref159] Negi G. Kumar A. Sharma S. S. (2011). Melatonin modulates neuroinflammation and oxidative stress in experimental diabetic neuropathy: effects on NF-kappaB and Nrf2 cascades. J. Pineal Res. 50, 124–131. doi: 10.1111/j.1600-079X.2010.00821.x, PMID: 21062351

[ref160] Nelson P. T. Smith C. D. Abner E. A. Schmitt F. A. Scheff S. W. Davis G. J.. (2009). Human cerebral neuropathology of type 2 diabetes mellitus. Biochim. Biophys. Acta 1792, 454–469. doi: 10.1016/j.bbadis.2008.08.005, PMID: 18789386 PMC2834412

[ref161] Neufeldt C. J. Cerikan B. Cortese M. Frankish J. Lee J. Y. Plociennikowska A.. (2022). SARS-CoV-2 infection induces a pro-inflammatory cytokine response through cGAS-STING and NF-kappaB. Commun. Biol. 5:45. doi: 10.1038/s42003-021-02983-5, PMID: 35022513 PMC8755718

[ref162] Nielsen S. Arnulf Nagelhus E. Amiry-Moghaddam M. Bourque C. Agre P. Petter Ottersen O. (1997). Specialized membrane domains for water transport in glial cells: high-resolution immunogold cytochemistry of aquaporin-4 in rat brain. J. Neurosci. 17, 171–180. doi: 10.1523/JNEUROSCI.17-01-00171.1997, PMID: 8987746 PMC6793699

[ref163] Niiya Y. Abumiya T. Yamagishi S. Takino J. Takeuchi M. (2012). Advanced glycation end products increase permeability of brain microvascular endothelial cells through reactive oxygen species-induced vascular endothelial growth factor expression. J. Stroke Cerebrovasc. Dis. 21, 293–298. doi: 10.1016/j.jstrokecerebrovasdis.2010.09.002, PMID: 21296593

[ref164] Nimmerjahn A. Kirchhoff F. Helmchen F. (2005). Resting microglial cells are highly dynamic surveillants of brain parenchyma *in vivo*. Science 308, 1314–1318. doi: 10.1126/science.1110647, PMID: 15831717

[ref165] Nishikawa T. Edelstein D. du X. L. Yamagishi S. I. Matsumura T. Kaneda Y.. (2000). Normalizing mitochondrial superoxide production blocks three pathways of hyperglycaemic damage. Nature 404, 787–790. doi: 10.1038/35008121, PMID: 10783895

[ref166] Nishioku T. Matsumoto J. Dohgu S. Sumi N. Miyao K. Takata F.. (2010). Tumor necrosis factor-alpha mediates the blood-brain barrier dysfunction induced by activated microglia in mouse brain microvascular endothelial cells. J. Pharmacol. Sci. 112, 251–254. doi: 10.1254/jphs.09292SC, PMID: 20118615

[ref167] Pajo A. T. Espiritu A. I. Apor A. Jamora R. D. G. (2021). Neuropathologic findings of patients with COVID-19: a systematic review. Neurol. Sci. 42, 1255–1266. doi: 10.1007/s10072-021-05068-7, PMID: 33483885 PMC7822400

[ref168] Pan J. Peng J. Li X. Wang H. Rong X. Peng Y. (2023). Transmission of NLRP3-IL-1beta signals in cerebral ischemia and reperfusion injury: from microglia to adjacent neuron and endothelial cells via IL-1beta/IL-1R1/TRAF6. Mol. Neurobiol. 60, 2749–2766. doi: 10.1007/s12035-023-03232-y, PMID: 36717480

[ref169] Pan L. Sze Y. H. Yang M. Tang J. Zhao S. Yi I.. (2022). Baicalein-a potent pro-homeostatic regulator of microglia in retinal ischemic injury. Front. Immunol. 13:837497. doi: 10.3389/fimmu.2022.837497, PMID: 35265083 PMC8899187

[ref170] Paolicelli R. C. Bolasco G. Pagani F. Maggi L. Scianni M. Panzanelli P.. (2011). Synaptic pruning by microglia is necessary for normal brain development. Science 333, 1456–1458. doi: 10.1126/science.1202529, PMID: 21778362

[ref171] Peerlings D. Bennink E. Dankbaar J. W. Velthuis B. K. Emmer B. J. Hoving J. W.. (2023). Standardizing the estimation of ischemic regions can harmonize CT perfusion stroke imaging. Eur. Radiol. 34, 797–807. doi: 10.1007/s00330-023-10035-1, PMID: 37572189 PMC10853359

[ref172] Peng X. Li C. Yu W. Liu S. Cong Y. Fan G.. (2020). Propofol attenuates hypoxia-induced inflammation in BV2 microglia by inhibiting oxidative stress and NF-kappaB/Hif-1alpha signaling. Biomed. Res. Int. 2020, 1–11. doi: 10.1155/2020/8978704 PMC7204316 32420378

[ref173] Puntener U. Booth S. G. Perry V. H. Teeling J. L. (2012). Long-term impact of systemic bacterial infection on the cerebral vasculature and microglia. J. Neuroinflammation 9:146. doi: 10.1186/1742-2094-9-146, PMID: 22738332 PMC3439352

[ref174] Qiu C. Sigurdsson S. Zhang Q. Jonsdottir M. K. Kjartansson O. Eiriksdottir G.. (2014). Diabetes, markers of brain pathology and cognitive function: the age Gene/environment susceptibility-reykjavik study. Ann. Neurol. 75, 138–146. doi: 10.1002/ana.24063, PMID: 24243491 PMC4540233

[ref175] Rashid I. Pathak A. K. Kumar R. Srivastava P. Singh M. Murali S.. (2019). Genome-wide comparative analysis of HIF binding sites in *Cyprinus Carpio* for in silico identification of functional hypoxia response elements. Front. Genet. 10:659. doi: 10.3389/fgene.2019.00659, PMID: 31379925 PMC6660265

[ref176] Rezaie P. Cairns N. J. Male D. K. (1997). Expression of adhesion molecules on human fetal cerebral vessels: relationship to microglial colonisation during development. Brain Res. Dev. Brain Res. 104, 175–189. PMID: 9466720 10.1016/s0165-3806(97)00153-3

[ref177] Rigato C. Buckinx R. Le-Corronc H. Rigo J. M. Legendre P. (2011). Pattern of invasion of the embryonic mouse spinal cord by microglial cells at the time of the onset of functional neuronal networks. Glia 59, 675–695. doi: 10.1002/glia.21140, PMID: 21305616

[ref178] Rutkai I. Mayer M. G. Hellmers L. M. Ning B. Huang Z. Monjure C. J.. (2022). Neuropathology and virus in brain of SARS-CoV-2 infected non-human primates. Nat. Commun. 13:1745. doi: 10.1038/s41467-022-29440-z, PMID: 35365631 PMC8975902

[ref179] Sadasivan S. Zanin M. O'Brien K. Schultz-Cherry S. Smeyne R. J. (2015). Induction of microglia activation after infection with the non-neurotropic a/CA/04/2009 H1N1 influenza virus. PLoS One 10:e0124047. doi: 10.1371/journal.pone.0124047, PMID: 25861024 PMC4393251

[ref180] Sadowska G. B. Chen X. Zhang J. Lim Y. P. Cummings E. E. Makeyev O.. (2015). Interleukin-1beta transfer across the blood-brain barrier in the ovine fetus. J. Cereb. Blood Flow Metab. 35, 1388–1395. doi: 10.1038/jcbfm.2015.134, PMID: 26082012 PMC4640327

[ref181] Sajja R. K. Green K. N. Cucullo L. (2015). Altered Nrf2 signaling mediates hypoglycemia-induced blood-brain barrier endothelial dysfunction in vitro. PLoS One 10:e0122358. doi: 10.1371/journal.pone.0122358, PMID: 25807533 PMC4373930

[ref182] Sajja R. K. Prasad S. Cucullo L. (2014). Impact of altered glycaemia on blood-brain barrier endothelium: an in vitro study using the hCMEC/D3 cell line. Fluids Barriers CNS 11:8. doi: 10.1186/2045-8118-11-8, PMID: 24708805 PMC3985548

[ref183] Salsman V. S. Chow K. K. H. Shaffer D. R. Kadikoy H. Li X. N. Gerken C.. (2011). Crosstalk between medulloblastoma cells and endothelium triggers a strong chemotactic signal recruiting T lymphocytes to the tumor microenvironment. PLoS One 6:e20267. doi: 10.1371/journal.pone.0020267, PMID: 21647415 PMC3103535

[ref184] Santos A. M. Calvente R. Tassi M. Carrasco M. C. Martín-Oliva D. Marín-Teva J. L.. (2008). Embryonic and postnatal development of microglial cells in the mouse retina. J. Comp. Neurol. 506, 224–239. doi: 10.1002/cne.21538, PMID: 18022954

[ref185] Schreck R. Rieber P. Baeuerle P. A. (1991). Reactive oxygen intermediates as apparently widely used messengers in the activation of the NF-kappa B transcription factor and HIV-1. EMBO J. 10, 2247–2258. doi: 10.1002/j.1460-2075.1991.tb07761.x, PMID: 2065663 PMC452914

[ref186] Schreibelt G. Kooij G. Reijerkerk A. Doorn R. Gringhuis S. I. Pol S.. (2007). Reactive oxygen species alter brain endothelial tight junction dynamics via rho a, PI3 kinase, and PKB signaling. FASEB J. 21, 3666–3676. doi: 10.1096/fj.07-8329com, PMID: 17586731

[ref187] Schurink B. Roos E. Radonic T. Barbe E. Bouman C. S. C. de Boer H. H.. (2020). Viral presence and immunopathology in patients with lethal COVID-19: a prospective autopsy cohort study. Lancet Microb. 1, e290–e299. doi: 10.1016/S2666-5247(20)30144-0, PMID: 33015653 PMC7518879

[ref188] Schwabenland M. Mossad O. Sievert A. Peres A. G. Ringel E. Baasch S.. (2023). Neonatal immune challenge poses a sex-specific risk for epigenetic microglial reprogramming and behavioral impairment. Nat. Commun. 14:2721. doi: 10.1038/s41467-023-38373-0, PMID: 37169749 PMC10175500

[ref189] Secrest A. M. Prince C. T. Costacou T. Miller R. G. Orchard T. J. (2013). Predictors of and survival after incident stroke in type 1 diabetes. Diab. Vasc. Dis. Res. 10, 3–10. doi: 10.1177/1479164112441006, PMID: 22535586 PMC3635676

[ref190] Shah M. A. Park D. J. Kang J. B. Kim M. O. Koh P. O. (2019). Baicalin attenuates lipopolysaccharide-induced neuroinflammation in cerebral cortex of mice via inhibiting nuclear factor kappa B (NF-kappaB) activation. J. Vet. Med. Sci. 81, 1359–1367. doi: 10.1292/jvms.19-0281, PMID: 31366818 PMC6785614

[ref191] Shah M. A. Park D. J. Kang J. B. Kim M. O. Koh P. O. (2020). Baicalin alleviates lipopolysaccharide-induced neuroglial activation and inflammatory factors activation in hippocampus of adult mice. Lab. Anim. Res. 36:32. doi: 10.1186/s42826-020-00058-w, PMID: 32983956 PMC7495851

[ref192] Sheikh M. H. Errede M. d'Amati A. Khan N. Q. Fanti S. Loiola R. A.. (2022). Impact of metabolic disorders on the structural, functional, and immunological integrity of the blood-brain barrier: therapeutic avenues. FASEB J. 36:e22107. doi: 10.1096/fj.202101297R 34939700

[ref193] Shen Y. Gu J. Liu Z. Xu C. Qian S. Zhang X.. (2018). Inhibition of HIF-1alpha reduced blood brain barrier damage by regulating MMP-2 and VEGF during acute cerebral ischemia. Front. Cell. Neurosci. 12:288. doi: 10.3389/fncel.2018.00288, PMID: 30233326 PMC6132021

[ref194] Shigemoto-Mogami Y. Hoshikawa K. Sato K. (2018). Activated microglia disrupt the blood-brain barrier and induce chemokines and cytokines in a rat in vitro model. Front. Cell. Neurosci. 12:494. doi: 10.3389/fncel.2018.00494, PMID: 30618641 PMC6300509

[ref195] Sobue K. Yamamoto N. Yoneda K. Hodgson M. E. Yamashiro K. Tsuruoka N.. (1999). Induction of blood-brain barrier properties in immortalized bovine brain endothelial cells by astrocytic factors. Neurosci. Res. 35, 155–164. doi: 10.1016/S0168-0102(99)00079-6, PMID: 10616919

[ref196] Song Y. Ding W. Bei Y. Xiao Y. Tong H. D. Wang L. B.. (2018). Insulin is a potential antioxidant for diabetes-associated cognitive decline via regulating Nrf2 dependent antioxidant enzymes. Biomed. Pharmacother. 104, 474–484. doi: 10.1016/j.biopha.2018.04.097, PMID: 29793180

[ref197] Song F. Hong X. Cao J. Ma G. Han Y. Cepeda C.. (2018). Kir 4.1 channels in NG2-glia play a role in development, potassium signaling, and ischemia-related myelin loss. Commun. Biol. 1:80. doi: 10.1038/s42003-018-0083-x, PMID: 30271961 PMC6123808

[ref198] Song H. Tomasevich A. Acheampong K. K. Schaff D. L. Shaffer S. M. Dolle J. P.. (2023). Detection of blood-brain barrier disruption in brains of patients with COVID-19, but no evidence of brain penetration by SARS-CoV-2. Acta Neuropathol. 146, 771–775. doi: 10.1007/s00401-023-02624-7, PMID: 37624381 PMC10592095

[ref199] Spranger J. Kroke A. Möhlig M. Hoffmann K. Bergmann M. M. Ristow M.. (2003). Inflammatory cytokines and the risk to develop type 2 diabetes: results of the prospective population-based European prospective investigation into Cancer and nutrition (EPIC)-Potsdam study. Diabetes 52, 812–817. doi: 10.2337/diabetes.52.3.812 12606524

[ref200] Stamatovic S. M. Keep R. F. Kunkel S. L. Andjelkovic A. V. (2003). Potential role of MCP-1 in endothelial cell tight junction 'opening': signaling via rho and rho kinase. J. Cell Sci. 116, 4615–4628. doi: 10.1242/jcs.00755, PMID: 14576355

[ref201] Stentz F. B. Umpierrez G. E. Cuervo R. Kitabchi A. E. (2004). Proinflammatory cytokines, markers of cardiovascular risks, oxidative stress, and lipid peroxidation in patients with hyperglycemic crises. Diabetes 53, 2079–2086. doi: 10.2337/diabetes.53.8.2079, PMID: 15277389

[ref202] Sun Y. Chen H. Dai J. Wan Z. Xiong P. Xu Y.. (2018). Glycyrrhizin protects mice against experimental autoimmune encephalomyelitis by inhibiting high-mobility group box 1 (HMGB1) expression and neuronal HMGB1 release. Front. Immunol. 9:1518. doi: 10.3389/fimmu.2018.01518, PMID: 30013568 PMC6036111

[ref203] Sun X. Zeng H. Wang Q. Yu Q. Wu J. Feng Y.. (2018). Glycyrrhizin ameliorates inflammatory pain by inhibiting microglial activation-mediated inflammatory response via blockage of the HMGB1-TLR4-NF-kB pathway. Exp. Cell Res. 369, 112–119. doi: 10.1016/j.yexcr.2018.05.012, PMID: 29763588

[ref204] Swinnen N. Smolders S. Avila A. Notelaers K. Paesen R. Ameloot M.. (2013). Complex invasion pattern of the cerebral cortex by microglial cells during development of the mouse embryo. Glia 61, 150–163. doi: 10.1002/glia.22421, PMID: 23001583

[ref205] Szalay G. Martinecz B. Lénárt N. Környei Z. Orsolits B. Judák L.. (2016). Microglia protect against brain injury and their selective elimination dysregulates neuronal network activity after stroke. Nat. Commun. 7:11499. doi: 10.1038/ncomms11499, PMID: 27139776 PMC4857403

[ref206] Tang R. Cao Q. Q. Hu S. W. He L. J. du P. F. Chen G.. (2022). Sulforaphane activates anti-inflammatory microglia, modulating stress resilience associated with BDNF transcription. Acta Pharmacol. Sin. 43, 829–839. doi: 10.1038/s41401-021-00727-z, PMID: 34272506 PMC8976037

[ref207] Tang Y. Su G. Li N. Li W. Chen G. Chen R.. (2019). Preventive agents for neurodegenerative diseases from resin of Dracaena cochinchinensis attenuate LPS-induced microglia over-activation. J. Nat. Med. 73, 318–330. doi: 10.1007/s11418-018-1266-y, PMID: 30426288

[ref208] Tao W. Zhang X. Ding J. Yu S. Ge P. Han J.. (2022). The effect of propofol on hypoxia- and TNF-alpha-mediated BDNF/TrkB pathway dysregulation in primary rat hippocampal neurons. CNS Neurosci. Ther. 28, 761–774. doi: 10.1111/cns.13809, PMID: 35112804 PMC8981449

[ref209] Thakur K. T. Miller E. H. Glendinning M. D. al-Dalahmah O. Banu M. A. Boehme A. K.. (2021). COVID-19 neuropathology at Columbia University Irving medical center/New York Presbyterian hospital. Brain 144, 2696–2708. doi: 10.1093/brain/awab148, PMID: 33856027 PMC8083258

[ref210] Threlkeld S. W. Lynch J. L. Lynch K. M. Sadowska G. B. Banks W. A. Stonestreet B. S. (2010). Ovine proinflammatory cytokines cross the murine blood-brain barrier by a common saturable transport mechanism. Neuroimmunomodulation 17, 405–410. doi: 10.1159/000288265, PMID: 20516722 PMC2914440

[ref211] Tsao C. W. Aday A. W. Almarzooq Z. I. Anderson C. A. M. Arora P. Avery C. L.. (2023). Heart disease and stroke Statistics-2023 update: a report from the American Heart Association. Circulation 147, e93–e621. doi: 10.1161/CIR.0000000000001123, PMID: 36695182 PMC12135016

[ref212] Tureyen K. Bowen K. Liang J. Dempsey R. J. Vemuganti R. (2011). Exacerbated brain damage, edema and inflammation in type-2 diabetic mice subjected to focal ischemia. J. Neurochem. 116, 499–507. doi: 10.1111/j.1471-4159.2010.07127.x, PMID: 21133923 PMC3076322

[ref213] Uddin M. A. Akhter M. S. Kubra K. T. Whitaker K. E. Shipley S. L. Smith L. M.. (2021). Hsp 90 inhibition protects the brain microvascular endothelium against oxidative stress. Brain Disord. 1:100001. doi: 10.1016/j.dscb.2020.100001, PMID: 33569547 PMC7869856

[ref214] Ungvari Z. Bailey-Downs L. Gautam T. Jimenez R. Losonczy G. Zhang C.. (2011). Adaptive induction of NF-E2-related factor-2-driven antioxidant genes in endothelial cells in response to hyperglycemia. Am. J. Physiol. Heart Circ. Physiol. 300, H1133–H1140. doi: 10.1152/ajpheart.00402.2010, PMID: 21217061 PMC3075025

[ref215] Vanlandewijck M. He L. Mäe M. A. Andrae J. Ando K. del Gaudio F.. (2018). A molecular atlas of cell types and zonation in the brain vasculature. Nature 554, 475–480. doi: 10.1038/nature25739, PMID: 29443965

[ref216] Vargas-Soria M. Garcia-Alloza M. Corraliza-Gomez M. (2023). Effects of diabetes on microglial physiology: a systematic review of in vitro, preclinical and clinical studies. J. Neuroinflammation 20:57. doi: 10.1186/s12974-023-02740-x, PMID: 36869375 PMC9983227

[ref217] Vittal Rao H. Bihaqi S. W. Iannucci J. Sen A. Grammas P. (2021). Thrombin signaling contributes to high glucose-induced injury of human brain microvascular endothelial cells. J. Alzheimers Dis. 79, 211–224. doi: 10.3233/JAD-200658, PMID: 33252072

[ref218] Wang Z. Leng Y. Tsai L. K. Leeds P. Chuang D. M. (2011). Valproic acid attenuates blood-brain barrier disruption in a rat model of transient focal cerebral ischemia: the roles of HDAC and MMP-9 inhibition. J. Cereb. Blood Flow Metab. 31, 52–57. doi: 10.1038/jcbfm.2010.195, PMID: 20978517 PMC3049473

[ref219] Wang X. Yu J. Y. Sun Y. Wang H. Shan H. Wang S. (2021). Baicalin protects LPS-induced blood-brain barrier damage and activates Nrf2-mediated antioxidant stress pathway. Int. Immunopharmacol. 96:107725. doi: 10.1016/j.intimp.2021.107725, PMID: 34162131

[ref220] Wanrooy B. J. Kumar K. P. Wen S. W. Qin C. X. Ritchie R. H. Wong C. H. Y. (2018). Distinct contributions of hyperglycemia and high-fat feeding in metabolic syndrome-induced neuroinflammation. J. Neuroinflammation 15:293. doi: 10.1186/s12974-018-1329-8, PMID: 30348168 PMC6198529

[ref221] Wen H. Tan J. Tian M. Wang Y. Gao Y. Gong Y. (2023). TGF-beta 1 ameliorates BBB injury and improves long-term outcomes in mice after ICH. Biochem. Biophys. Res. Commun. 654, 136–144. doi: 10.1016/j.bbrc.2023.03.007, PMID: 36931108

[ref222] Wilhelmsen K. Mesa K. R. Prakash A. Xu F. Hellman J. (2012). Activation of endothelial TLR2 by bacterial lipoprotein upregulates proteins specific for the neutrophil response. Innate Immun. 18, 602–616. doi: 10.1177/1753425911429336, PMID: 22186927 PMC3444510

[ref223] Wright G. J. Jones M. Puklavec M. J. Brown M. H. Barclay A. N. (2001). The unusual distribution of the neuronal/lymphoid cell surface CD200 (OX2) glycoprotein is conserved in humans. Immunology 102, 173–179. doi: 10.1046/j.1365-2567.2001.01163.x, PMID: 11260322 PMC1783166

[ref224] Wu K. C. Cui J. Y. Klaassen C. D. (2012). Effect of graded Nrf2 activation on phase-I and -II drug metabolizing enzymes and transporters in mouse liver. PLoS One 7:e39006. doi: 10.1371/journal.pone.0039006, PMID: 22808024 PMC3395627

[ref225] Wu S. Y. Pan B. S. Tsai S. F. Chiang Y. T. Huang B. M. Mo F. E.. (2020). BDNF reverses aging-related microglial activation. J. Neuroinflammation 17:210. doi: 10.1186/s12974-020-01887-1, PMID: 32664974 PMC7362451

[ref226] Xing C. Li W. Deng W. Ning M. Lo E. H. (2018). A potential gliovascular mechanism for microglial activation: differential phenotypic switching of microglia by endothelium versus astrocytes. J. Neuroinflammation 15:143. doi: 10.1186/s12974-018-1189-2, PMID: 29764475 PMC5952884

[ref227] Xing J. Lu J. (2016). HIF-1alpha activation attenuates IL-6 and TNF-alpha pathways in Hippocampus of rats following transient global ischemia. Cell. Physiol. Biochem. 39, 511–520. doi: 10.1159/000445643, PMID: 27383646

[ref228] Xu X. Yang M. Zhang B. Dong J. Zhuang Y. Ge Q.. (2023). HIF-1alpha participates in secondary brain injury through regulating neuroinflammation. Trans. Neurosci. 14:20220272. doi: 10.1515/tnsci-2022-0272, PMID: 36815939 PMC9921917

[ref229] Ya B. L. Li H. F. Wang H. Y. Wu F. Xin Q. Cheng H. J.. (2017). 5-HMF attenuates striatum oxidative damage via Nrf2/ARE signaling pathway following transient global cerebral ischemia. Cell Stress Chaperones 22, 55–65. doi: 10.1007/s12192-016-0742-0, PMID: 27812888 PMC5225060

[ref230] Yan J. J. Du G. H. Qin X. M. Gao L. (2020). Baicalein attenuates the neuroinflammation in LPS-activated BV-2 microglial cells through suppression of pro-inflammatory cytokines, COX2/NF-kappaB expressions and regulation of metabolic abnormality. Int. Immunopharmacol. 79:106092. doi: 10.1016/j.intimp.2019.106092, PMID: 31863920

[ref231] Yan J. Zhang Z. Shi H. (2012). HIF-1 is involved in high glucose-induced paracellular permeability of brain endothelial cells. Cell. Mol. Life Sci. 69, 115–128. doi: 10.1007/s00018-011-0731-5, PMID: 21617913 PMC11115066

[ref232] Yang T. T. Lin C. Hsu C. T. Wang T. F. Ke F. Y. Kuo Y. M. (2013). Differential distribution and activation of microglia in the brain of male C57BL/6J mice. Brain Struct. Funct. 218, 1051–1060. doi: 10.1007/s00429-012-0446-x, PMID: 22886465

[ref233] Yang Y. Salayandia V. M. Thompson J. F. Yang L. Y. Estrada E. Y. Yang Y. (2015). Attenuation of acute stroke injury in rat brain by minocycline promotes blood-brain barrier remodeling and alternative microglia/macrophage activation during recovery. J. Neuroinflammation 12:26. doi: 10.1186/s12974-015-0245-4, PMID: 25889169 PMC4340283

[ref234] Yang X. Xu Y. Gao W. Wang L. Zhao X. Liu G.. (2022). Hyperinsulinemia-induced microglial mitochondrial dynamic and metabolic alterations lead to neuroinflammation in vivo and in vitro. Front. Neurosci. 16:1036872. doi: 10.3389/fnins.2022.1036872, PMID: 36466168 PMC9709447

[ref235] Yao X. Zhao J. Yuan Y. Wang C. Yu Z. Huang Z.. (2023). Prolonged early exposure to a high-fat diet augments the adverse effects on Neurobehavior and hippocampal neuroplasticity: involvement of microglial insulin signaling. Am. J. Pathol. 193, 1568–1586. doi: 10.1016/j.ajpath.2023.06.005, PMID: 37356575

[ref236] Yousef H. Czupalla C. J. Lee D. Chen M. B. Burke A. N. Zera K. A.. (2019). Aged blood impairs hippocampal neural precursor activity and activates microglia via brain endothelial cell VCAM1. Nat. Med. 25, 988–1000. doi: 10.1038/s41591-019-0440-4, PMID: 31086348 PMC6642642

[ref237] Yu T. Jhun B. S. Yoon Y. (2011). High-glucose stimulation increases reactive oxygen species production through the calcium and mitogen-activated protein kinase-mediated activation of mitochondrial fission. Antioxid. Redox Signal. 14, 425–437. doi: 10.1089/ars.2010.3284, PMID: 20518702 PMC3025178

[ref238] Yu Z. Lin L. Jiang Y. Chin I. Wang X. Li X.. (2019). Recombinant FGF21 protects against blood-brain barrier leakage through Nrf2 upregulation in type 2 diabetes mice. Mol. Neurobiol. 56, 2314–2327. doi: 10.1007/s12035-018-1234-2, PMID: 30022432 PMC6339597

[ref239] Zelic M. Pontarelli F. Woodworth L. Zhu C. Mahan A. Ren Y.. (2021). RIPK1 activation mediates neuroinflammation and disease progression in multiple sclerosis. Cell Rep. 35:109112. doi: 10.1016/j.celrep.2021.109112, PMID: 33979622 PMC8917516

[ref240] Zeng J. Chen Y. Ding R. Feng L. Fu Z. Yang S.. (2017). Isoliquiritigenin alleviates early brain injury after experimental intracerebral hemorrhage via suppressing ROS- and/or NF-kappaB-mediated NLRP3 inflammasome activation by promoting Nrf2 antioxidant pathway. J. Neuroinflammation 14:119. doi: 10.1186/s12974-017-0895-5, PMID: 28610608 PMC5470182

[ref241] Zhang L. Luo X. Chen F. Yuan W. Xiao X. Zhang X.. (2018). Lnc RNA SNHG1 regulates cerebrovascular pathologies as a competing endogenous RNA through HIF-1alpha/VEGF signaling in ischemic stroke. J. Cell. Biochem. 119, 5460–5472. doi: 10.1002/jcb.26705, PMID: 29377234

[ref242] Zhang L. Nair A. Krady K. Corpe C. Bonneau R. H. Simpson I. A.. (2004). Estrogen stimulates microglia and brain recovery from hypoxia-ischemia in normoglycemic but not diabetic female mice. J. Clin. Invest. 113, 85–95. doi: 10.1172/JCI200418336, PMID: 14702112 PMC300764

[ref243] Zhang H. Sumbria R. K. Chang R. Sun J. Cribbs D. H. Holmes T. C.. (2023). Erythrocyte-brain endothelial interactions induce microglial responses and cerebral microhemorrhages in vivo. J. Neuroinflammation 20:265. doi: 10.1186/s12974-023-02932-5, PMID: 37968737 PMC10647121

[ref244] Zhang Y. Wang Y. Zuo Z. Wang Z. Roy J. Hou Q.. (2014). Effects of tissue plasminogen activator timing on blood-brain barrier permeability and hemorrhagic transformation in rats with transient ischemic stroke. J. Neurol. Sci. 347, 148–154. doi: 10.1016/j.jns.2014.09.036, PMID: 25292413

[ref245] Zhao Y. Fu B. Zhang X. Zhao T. Chen L. Zhang J.. (2014). Paeonol pretreatment attenuates cerebral ischemic injury via upregulating expression of pAkt, Nrf2, HO-1 and ameliorating BBB permeability in mice. Brain Res. Bull. 109, 61–67. doi: 10.1016/j.brainresbull.2014.09.008, PMID: 25286445

[ref246] Zhao Z. Huang G. Wang B. Zhong Y. (2013). Inhibition of NF-kappaB activation by Pyrrolidine dithiocarbamate partially attenuates hippocampal MMP-9 activation and improves cognitive deficits in streptozotocin-induced diabetic rats. Behav. Brain Res. 238, 44–47. doi: 10.1016/j.bbr.2012.10.018 23089644

[ref247] Zhong L. D'Urso A. Toiber D. Sebastian C. Henry R. E. Vadysirisack D. D.. (2010). The histone deacetylase Sirt6 regulates glucose homeostasis via Hif1alpha. Cell 140, 280–293. doi: 10.1016/j.cell.2009.12.041, PMID: 20141841 PMC2821045

[ref248] Zille M. Ikhsan M. Jiang Y. Lampe J. Wenzel J. Schwaninger M. (2019). The impact of endothelial cell death in the brain and its role after stroke: a systematic review. Cell Stress 3, 330–347. doi: 10.15698/cst2019.11.203, PMID: 31799500 PMC6859425

[ref249] Zrzavy T. Höftberger R. Berger T. Rauschka H. Butovsky O. Weiner H.. (2019). Pro-inflammatory activation of microglia in the brain of patients with sepsis. Neuropathol. Appl. Neurobiol. 45, 278–290. doi: 10.1111/nan.12502, PMID: 29804289 PMC6487964

